# Student well-being in times of COVID-19 in the Netherlands: basic psychological need satisfaction and frustration within the academic learning environment

**DOI:** 10.1007/s10212-023-00680-x

**Published:** 2023-03-02

**Authors:** L. Kiltz, M. Trippenzee, J. Fleer, M. Fokkens-Bruinsma, E. P. W. A. Jansen

**Affiliations:** 1 Department Teacher Education, Behavioural and Social Sciences Faculty, University of Groningen, Groningen, The Netherlands; 2 Department Health Psychology, Faculty of Medical Sciences, University Medical Center Groningen, Groningen, The Netherlands

**Keywords:** Student well-being, Mixed-method study, Self-determination theory, Academic learning environment, COVID-19, University

## Abstract

The COVID-19 pandemic affected student well-being through measures such as closing educational institutions and social distancing, which forced universities to adapt the student learning environment. Previous research has demonstrated that the learning environment influences student well-being by satisfying their basic psychological needs of autonomy, competence, and relatedness. The present study therefore aimed to investigate, against the background of the pandemic, (1) how the basic psychological needs related to student well-being, (2) how students perceived interaction within the academic system, and (3) how they would like the “new educational normal” to look. To address these aims, we implemented a cross-sectional survey which included both quantitative measures and qualitative open-ended questions and distributed it at a Dutch university (*n* = 653). To identify the predictive strength of need satisfaction and frustration, we ran multiple regressions. We found that need satisfaction and frustration were significantly related to the well-being measures, of which relatedness was only weakly related to student well-being. Moreover, students reported restricted interactions with their teachers and fellow students, leading to feelings of disconnectedness, as well as struggles to establish or maintain relationships. In contrast with the quantitative findings, students highlighted relatedness as an essential, currently lacking part of their experience. They suggested a potential hybrid learning environment with a focus on social cohesion. This study emphasizes the relevance of interpersonal connections within higher education and provides practical ideas for post-pandemic academic structures. These insights can support systemic investments of universities in student well-being and creating a healthier learning environment for the future.

**Supplementary Information:**

The online version contains supplementary material available at 10.1007/s10212-023-00680-x.

## Introduction

By 2022, it has become clear that the COVID-19 pandemic has considerably impacted the psychological well-being of the entire population and of university students in particular. The pandemic has not only resulted in direct effects such as sickness and death, but also measures to mitigate the spread including social distancing, quarantine, and lockdown, which have also taken a psychological toll. However, these measures may have affected specific populations more than others. For example, closing educational institutions has been found to have substantially impacted university students (e.g., Cao et al., 2020; Gautam & Sharma, 2020). The sudden restrictions on the educational system, which led to remote and online teaching, revealed mismatches between students’ needs and the academic world, many of which were present before the pandemic (Nandy et al., 2021). In this sense, the COVID-19 pandemic can be considered a magnifying glass, bringing to the forefront issues in academia that must be addressed within as well as beyond the scope of a global crisis. Educational systems tend to reorganize in times of crisis, providing new opportunities and leading to a “new educational normal” (Geertsema & Bolander Laksov, 2019; Yang, 2020). We propose that remote teaching has compromised need satisfaction in particular, which has in turn affected student well-being. Therefore, understanding how universities can satisfy students’ needs can help in the development of a more satisfactory environment in the post-pandemic world. To this end, we investigate how the changes within the academic learning environment (ALE) have affected students’ well-being, how students perceive interactions with others at the university, and how they envision the new educational normal.

### Student well-being during COVID-19

The COVID-19 pandemic has continuously affected students’ well-being in all its sub-facets,^1^ as particularly younger people seem to struggle with the associated psychological burdens (Dopmeijer et al., 2021; Stevens et al., 2020). Research focused on the periods shortly before and after the beginning of the pandemic indicates a significant decrease in student well-being and specifically social well-being (Hagemeier & Dowling-McClay, 2020; Pierce et al., 2020). Even before the pandemic, students struggled more psychologically than their peers outside of academia (Stallman, 2010), which could indicate that the ALE affects the well-being of this group. COVID-19 and resulting changes within the ALE might have exacerbated this effect. Taylor (2019) mentions uncertainty, disruption of routines, financial insecurity, separation from close people, and social isolation as pandemic-related stressors, all of which are characteristic of student life too: leaving behind the security of one’s home, adjusting to the flexibility of the university, coping with a financially precarious situation, and building a new social network. These factors considerably affect student well-being—even without a global pandemic.

### Students’ well-being and the academic learning environment

Researchers define various social and academic contextual factors within the ALE that affect students’ well-being (Baik et al., 2019; Chang et al., 2021). For example, social contextual factors include institutional, social, and peer support (Byrd & McKinney, 2012; Tao et al., 2000), and academic factors include university services (Chang et al., 2021; Sharp & Theiler, 2018) and performance-related aspects, such as setting academic requirements and appropriate workloads (Byrd & McKinney, 2012). Beyond these factors, students’ satisfaction with the campus climate and their sense of belonging appear essential for their well-being (Baik et al., 2019; Byrd & McKinney, 2012). The COVID-19 pandemic profoundly affected the ALE and its stakeholders (Nandy et al., 2021; Watermeyer et al., 2021; Yang, 2020). For instance, many students reacted negatively to the initial switch to online teaching (Besser et al., 2020), and social distancing measures worsened social relationships with fellow students and teachers, resulting in an impaired sense of belonging (Crawford & Stone, 2020; Pelikan et al., 2021).

#### Satisfying basic psychological needs within the learning environment

The social and academic contextual factors elaborated herein resonate with the concept of the basic psychological needs (BPN) of autonomy, competence, and relatedness. According to self-determination theory (Deci & Ryan, 1985), satisfying BPN leads to heightened well-being. Need satisfaction also has been associated with greater well-being in student populations (Sulea et al., 2015; Van den Broeck et al., 2008) and, during COVID-19, the general population (Cantarero et al., 2020; Šakan et al., 2020). In educational contexts, BPN influence students’ well-being through contextual and interpersonal aspects within the ALE (Kiltz et al., 2020; Niemiec & Ryan, 2009; Stanton et al., 2016). Due to COVID-19, an altered ALE may have compromised need satisfaction and, in turn, well-being.

*Autonomy* describes the sense of freedom and control over one’s own actions, unaffected by external controls (Deci & Ryan, 1987). In education, promoting students’ participatory voice, flexibility, and feelings of personal relevance appears to stimulate their feelings of autonomy (Niemiec & Ryan, 2009; Kiltz et al., 2020; Stanton et al., 2016). However, pandemic-related stressors such as uncertainty and losing daily routines may compromise autonomy by restricting students’ control (Besser et al., 2020). It should be mentioned that attending university already entails new, unknown structures and choices, as well as previously unexperienced levels of independence, so these stressors are present regardless. During the pandemic, uncertainty and unstable daily routines may have further contributed to students’ autonomy losses and gains: Whereas externally imposed measures, loss of control, and uncertainty undermine students’ sense of autonomy, leading to heightened anxiety (Cao et al., 2020), students reportedly also appreciated the flexibility and autonomy that came with distance education (Boling et al., 2012; Lall & Singh, 2020).

*Competence* relates to the belief that the efforts one invests in a task will lead to the intended outcome (Deci & Ryan, 1987). At university, providing helpful feedback and creating optimally challenging tasks within a course promotes students’ sense of competence (Niemiec & Ryan, 2009; Stanton et al., 2016). University closures during a global pandemic could deprive students of feelings of competence: Students have reported remote teaching practices during COVID-19 as less effective in terms of learning (Gautam & Sharma, 2020; Stevens et al., 2020). Remote education also comes with adverse academic outcomes, including detaching, demotivation, and distress (Crawford & Stone, 2020; Stevens et al., 2020). Pelikan et al. (2021) unsurprisingly show that students’ sense of competence determines whether they procrastinated or persevered during the pandemic, which likely influenced their well-being and academic performance.

*Relatedness* pertains to a feeling of being connected to a specific social group or institution (Deci & Ryan, 2000). In higher education, giving students a feeling of being acknowledged, respected, and supported is beneficial for their academic success and well-being (Kiltz et al., 2020; Niemiec & Ryan, 2009). Current research emphasizes the informal side of teacher–student relationships such as warmth and attachment (Tormey, 2021). During the COVID-19 pandemic, however, relatedness has been the most obviously affected BPN. Inevitably, social distancing measures created social disruption and greatly affected well-being (e.g., Ford, 2021). Again, students may have been at particular risk, as they already had to socially readjust at the beginning of their studies. Accordingly, students’ sense of relatedness appeared compromised during the pandemic, which affected their learning outcomes (Crawford & Stone, 2020; Pelikan et al., 2021). At the same time, students with a greater sense of belonging appeared to adapt better to the sudden changes within the ALE (Besser et al., 2020). Moreover, being socially supported and connected can prevent mental problems and academic dropouts in remote ALEs (Cao et al., 2020; Crawford & Stone, 2020). Taken together, pandemic-related changes within the ALE have influenced students’ need satisfaction and thus their well-being.

### Research aims

**Fig. 1:**
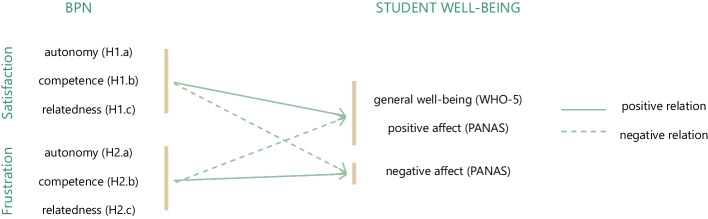
Visualization of hypotheses 1 and 2 and the relevant variables

Using a mixed-method design, we aimed to disentangle the psychological impact of the COVID-19 crisis and subsequent measures on university student well-being in relation to their ALE. In addition, we investigated whether and how the aforementioned BPN influenced student well-being 1 year into the pandemic, beyond sociodemographic and study-related variables (see Fig. 1). Moreover, considering the extent to which the ALE has been affected, we propose that perceptions of what constitutes good teaching have shifted. Therefore, we are also interested in students’ interaction experiences and their perceptions of an educational new normal. In summary, the present study focuses on the following research aims^2^:

- RQ.1 How has the COVID-19 pandemic, specifically considering the BPN as factors within the ALE, influenced student well-being one year into the pandemic?
  - H1.a–c Satisfaction of the BPN of autonomy (a), competence (b), and relatedness (c) relates to heightened student well-being in the form of higher general well-being as well as more frequent positive and less frequent negative affect in the context of COVID-19.
  - H2.a–c Frustration of the BPN of autonomy (a), competence (b), and relatedness (c) relates to lower student well-being in the form of lower general well-being as well as less frequent positive and more frequent negative affect in the context of COVID-19.
- RQ. 2 How did students perceive interactions with teachers and fellow students in the context of COVID-19?
- RQ. 3 What are students’ ideas for an educational new normal?

We drew on both quantitative and qualitative data to answer these research questions; however, because we used different analysis approaches for each type of data, we report them separately and then provide an integrated conclusion.

## Method

We opted for a cross-sectional, mixed-method survey design to explore the research questions and hypotheses. The ethical committee at the university approved the study procedure,^3^ and we preregistered it with the Open Science Framework.^4^

### Sample

The sample consisted of students primarily enrolled within four faculties at the university under study: medical; social and behavioral; spatial; and science, technology, engineering, and mathematics (STEM) faculties. Of the initial 918 students contacted, we excluded those who filled in less than half of the questionnaire (*n* = 255) and those who did not vary in their response (*n* = 10), resulting in a final sample size of 653 students. This sample included 371 bachelor students (56.8%), 254 master students (38.9%), and 27 pre-master students^5^ (4.1%). Of these students, 62.2% identified as female, 36.1% as male, and 1.7% as other or preferred not to say. Participants in the sample ranged in age from 17 to 44 years (*M* = 22.1 years, *SD* = 2.8). Moreover, 20.7% of students lived alone, and the remaining students lived together with housemates, family, or partners. Finally, 156 students (23.9%) were international, and 229 (35.1%) were first-generation students.

### Data collection

**Fig. 2:**
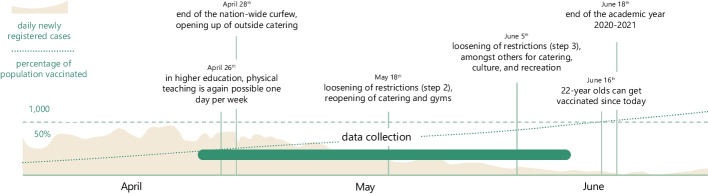
Study timeline, including the relevant pandemic events*.* Note. All societal and pandemic developments pertain to the Netherlands. All university events concern the university in question. Sources: coronavirus.nl, nos.nl, rijksoverheid.nl, rivm.nl

The data collection took place about a year into the pandemic in the Netherlands (Fig. 2). The participating faculties distributed a link to the survey among their students. In addition, we posted the questionnaire on social media. Before the participants started the survey, they consented to voluntary participation and anonymized open data publication. The questionnaire was available in English and Dutch and accessible via Qualtrics (see the supplementary information^6^). After collecting sociodemographic and academic information, we assessed students’ well-being and ALE perception. Participation took about 20 min, and participants received an informative brochure about how to care for one’s well-being during the pandemic. As an incentive for the faculties, the researchers provided faculty-specific reports including preliminary results.

### Measures

We assessed well-being using two scales. First, the WHO-5 Well-Being Index (World Health Organization, 1998) measures participants’ general well-being. Because we aimed to investigate participants’ current experiences, students rated how often they encountered well-being–related aspects over the previous two weeks. This measure also included the Positive and Negative Affect Schedule (PANAS; Watson et al., 1988) to measure students’ emotional well-being (i.e., extent to which students experienced positive and negative emotions) over the previous two weeks. Second, we included the Basic Psychological Need Satisfaction and Frustration Scale (Chen et al., 2015) to assess need satisfaction and frustration. In line with the researchers responsible for the original scale, we adjusted the scale, such that statements are applied to the context of studying in relation to students’ ALE. An exploratory factor analysis largely supported a three-factor solution, one for each need. Finally, participants could provide opinions and explain their experiences in more detail in open-ended questions—namely, how they would describe their interactions with teachers and fellow students and how they would shape their educational new normal.

### Analysis

For the quantitative analysis, we used IBM SPSS 26. Before undertaking the analysis, we omitted outliers and checked for potential sociodemographic control variables: students’ gender, age, living situation, nationality, first-generation status, faculty, and study phase. To this end, we used correlational analysis and multivariate analysis of variance (MANOVA). Subsequently, we correlated well-being and need measures. To investigate the variable’s predictive power, we ran two-model multiple regressions with forced entry for each of the three outcome measures: general well-being (WHO-5), positive affect, and negative affect (PANAS). The first model comprised sociodemographic variables significantly associated with well-being or need measures; the second also encompassed need satisfaction and frustration.

We analyzed the students’ qualitative answers without knowing their sociodemographic or well-being data. Low-level interpretation analysis methods such as thematic analysis or content analysis were most appropriate for our purposes (Vaismoradi et al., 2013). Therefore, we analyzed the qualitative data obtained from the three open-ended questions using a content analysis approach to assess their “what” and “how” (Elo & Kyngäs, 2008). Such an approach supports qualitative analysis while also quantifying the data to search for common themes and issues (Vaismoradi et al., 2013). In line with this approach, we created codes that emerged from the data throughout the analysis and subsequently formed overarching themes based on clusters of these codes.

## Results

### Students’ well-being and BPN

#### Well-being, sociodemographic, and study-related variables

We calculated the means and standard deviations of well-being and the BPN measures (see Table 1). Using Topp et al.’s (2015) WHO-5 cut-off score of 12.5 as a threshold differentiating between indicating good (if above) and poor well-being (if below), we found that 69.7% of the sample (*n* = 455) displayed poor well-being. Regarding potential control variables, the following elements related significantly to students’ well-being or BPN: gender, age, living situation, nationality, first-generation student status, and faculty. Regarding the latter, our MANOVA indicated that, on average, students at the medical faculty scored significantly higher than students of other faculties for positive affect and need satisfaction. In contrast, students at the STEM faculty scored significantly lower than students of other faculties in terms of negative affect, competence, and relatedness frustration. Therefore, we included dummy variables for the faculties in the multiple regression analyses, with the STEM faculty as the largest representation in our sample as a reference.

**Table 1:** Means (M) and standard deviations (SD) of the well-being and need satisfaction and frustration variables

Measure	Range (min–max)	*M*	*SD*
Well-being			
WHO-5	0–25	9.7	5.0
Positive affect	10–50	26.0	7.6
Negative affect		23.9	8.2
Need satisfaction and frustration			
Autonomy satisfaction	1–5	3.2	0.8
Autonomy frustration		3.3	0.9
Competence satisfaction		3.2	0.8
Competence frustration		2.9	1.1
Relatedness satisfaction		3.5	0.9
Relatedness frustration		2.2	0.8

#### Need satisfaction and frustration

Table 2 depicts the correlations between students’ well-being and BPN. All measures surrounding need satisfaction and frustration significantly correlated with the well-being measures in the hypothesized directions. While the competence measures correlated strongly with well-being outcomes, the relatedness measures correlated more weakly. Subsequently, we ran the two multiple regressions for each well-being variable (see Table 3). Entering the BPN in the second model substantially increased the explained variance: The second models accounted for approximately 50% of variation, whereas the first models explained only around 6%. These results emphasize the added value of the BPN beyond sociodemographic and study-related variables.

**Table 2:**
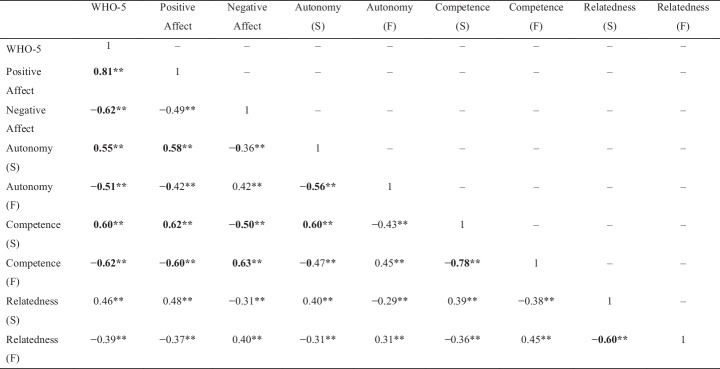
Correlations among the well-being and need satisfaction and frustration variables

*p < 0.05, **p < 0.01; correlations above 0.50 are considered large effect sizes (Cohen, 1992) and are displayed in bold. *S* satisfaction, *F* frustration

**Table 3:** Summary of the multiple regressions with WHO-5, positive affect, and negative affect as the outcome variables, students’ need satisfaction and frustration as predictors, and their sociodemographics as control variables

	WHO-5		Positive Affect		Negative Affect	
	Model 1	Model 2	Model 1	Model 2	Model 1	Model 2
Predictors	*β*	*β*	*β*	*β*	*β*	*β*
Gender (male : female)	−.01	.01	.01	.03	.16**	.11**
Nationality (int : Dutch)	.16**	.10**	.13**	.10**	−.15**	−.06
Living alone (yes : no)	.02	.03	.01	−.00	.05	.04
First gen (yes : no)	.02	.01	.04	.04	.04	.05
Age	−.10*	−.05	−.11*	−.04	.12**	.09**
Faculty (STEM: SoS)	.07	.06*	.04	.01	−.07	−.05
Faculty (STEM : M)	.20**	.09**	.19**	.05	−.15**	−.06
Faculty (STEM: SpS)	.08	.06	.02	−.01	−.14**	−.09**
Faculty (STEM : other)	.04	.04	.03	.03	−.02	.01
Faculty (STEM : ≥ 2F)	.01	.03	.04	.05	.05	.03
Autonomy (S)		.17**		.25**		.01
Autonomy (F)		−.20**		−.05		.16**
Competence (S)		.13**		.20**		−.04
Competence (F)		−.27**		−.23**		.44**
Relatedness (S)		.18**		.21**		**−**.00
Relatedness (F)		.03		.05		.12**
Adjusted *R*^*2*^	.06	.52	.05	.51	.07	.45
*F*	4.50	40.90	3.83	38.83	5.71	30.75

for dichotomous variables, the values are 0:1 for the two categories; *int* international student, *First gen* first-generation student, *SoS* affiliation with the social science faculty, *M* affiliation with medical faculty, *SpS* affiliation with spatial science faculty, *STEM faculty* affiliation with STEM faculty, *other* affiliation with another than these four faculties, ≥ *2F* affiliation with more than one faculty, *S* satisfaction, *F* frustration

Except for relatedness frustration, all need measures significantly predicted students’ general well-being, with satisfaction leading to greater and frustration to lesser well-being. Of these variables, competence frustration constituted the strongest and competence satisfaction the weakest predictors. Furthermore, students’ nationality and affiliation with the medical or social faculty, compared with the STEM faculty, remained significant predictors when we entered the BPN variables. Dutch students reported greater well-being than international students, and students studying at the medical and social faculties rated their well-being as greater than STEM faculty students.

For positive affect, all three need satisfaction measures exhibited positive predictive strength, with autonomy as the strongest predictor. Of the frustration measures, only competence frustration constituted a significant predictor and led to lower positive affect. Next to the BPN, solely students’ nationality predicted positive affect. Again, Dutch students experienced positive affect more frequently than international students.

Finally, only need frustration significantly predicted students’ negative affect, with greater frustration resulting in more negative affect. Competence frustration was the strongest predictor; relatedness frustration was the weakest. Beyond the BPN, students’ gender, age, and affiliation with the spatial science faculty compared to the STEM faculty significantly influenced negative affect. Female and older students experienced negative affect more frequently, and students studying at the spatial science faculty experienced negative affect less frequently than those at the STEM faculty. Consequently, we can confirm H1 only for general well-being and positive affect as positive well-being measures, not for negative affect. In contrast, our findings confirm H2 only for the negative well-being measure of negative affect and less coherently for the positive ones of general well-being and positive affect. In particular, relatedness frustration remained insignificant.

### Students’ experiences

We report the main findings per question grouped into themes. We do not include 12.9% of responses pertaining to interactions and 24.5% of those related to the new normal, due to missing, unclear, or uninterpretable answers.

#### Students’ interaction with teachers

##### Negative and positive aspects of the interaction

Most students described interactions with their teachers as nonexistent or restricted, using terms such as “stressful,” “cold,” and “impersonal.” Online education and asynchronous teaching inevitably hindered these interactions with teachers and the ability to address issues other than the focal course content. Hence, students perceived interactions as one-sided and solely content-related. In contrast, fewer students described the interaction with their teachers as good and supportive. The students attributed positive aspects to teachers’ practices such as doing their best by making time for students or implementing creative teaching methods, as illustrated when a student stated, “They are always willing to help, and I do not hesitate to contact them when I need to.”

##### Teachers’ impact on interaction

Teachers’ efforts in their teaching activities accounted for the quality of interactions. Students indicated that they did not appreciate teachers who uploaded online lectures that were already recorded before COVID-19 but did appreciate innovative teaching methods to get them involved (e.g., using Gathertown, creating podcasts or knowledge clips). Moreover, students experienced interaction with teachers and mentors who engaged in one-on-one contact as better or more supportive than interactions with teachers who did not do so.

##### Sense of belonging

Teaching online caused students to feel unseen and disconnected from their teachers. Feeling disconnected appeared to relate to class size, particularly in relatively large classes taught online. This lack of sense of belonging affected how students progressed in their studies and how approachable they perceived their teachers to be, as one student stated:

I feel unconnected to what is going on in class, and as such, it is difficult to engage with the teacher and the material. As a result, I zone out for substantial parts of classes, and I feel much less inclined to approach teachers with questions or other talk, which is something I would frequently do in and after on-site classes.

#### Students’ interaction with fellow students

##### Negative and positive aspects regarding interactions

Just as with teachers, most students reported restricted or no interactions with fellow students, mainly due to social distancing, online teaching, and few in-person events. They characterized the interaction as distant, impersonal, or superficial and expressed regret; one student explained: “I do not have any contact with fellow students. Studying has become very individual, unfortunately.” Conversely, fewer students perceived interactions as good, supportive, or pleasant; only those who had previous onsite teaching perceived interactions with their fellow students positively. Furthermore, good interactions were associated with the feeling of relatedness and common humanity, referred to as a sense of “being in it together.” A student emphasized this aspect: “[You] notice that many students feel the need to connect with each other and talk about their study.”

##### Group assignments

Group assignments were frequently reported as an aspect of peer-to-peer interactions. On the one hand, students perceived such group work as helpful for meeting up with fellow students. On the other hand, group assignments seemed more forced and uncomfortable in an online setting. Respondents mentioned difficulties such as being less spontaneous in the conversational flow, struggling with technical issues, and focusing on the content instead of personal matters.

##### Getting to know people

Particularly first-year students, international students, and students who switched programs reported that they could not get to know their fellow students properly due to the online environment. Their fellow students felt like strangers they had never met in person. One first-year student confirmed, “The little interactions I’ve had have been fun. Other than that, as a freshman, I don’t really know anyone at all.” In addition, students who did get to know their course mates reported feeling unrelated and disconnected. Significant aspects of onsite teaching such as having breaks together were lacking, so building sustainable relationships appeared complicated. Although the student associations’ activities seemed helpful in getting to know others, students also tried to create opportunities on their own to meet: “We have now also sat in the park with some students from a distance, to get to know each other in a different way and away from the study stress. Nice initiative from a fellow student.”

##### Higher quality, lower quantity

Students frequently differentiated friends they had made before the pandemic and those with whom they did not, noting that prior friendships seemed to be of higher quality. Consequently, students appreciated these stronger bonds, which helped and supported them throughout the pandemic. However, students still felt they had fewer relationships than during pre-COVID-19 times, which they considered a pity.

#### Students’ ideas for an educational “new normal”

##### Onsite, hybrid, or online teaching

**Fig. 3:**
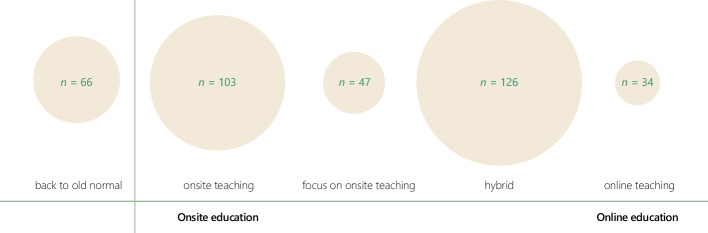
Clusters of answers regarding what students want for a new educational normal. Note. The larger the bubble, the more students answered according to the cluster. Students could have mentioned more than one aspect in their answers

Figure 3 illustrates the range of educational settings that students mentioned as their preferred future ALE. Several students stated that they did not want any educational new normal and preferred the “old normal,” emphasizing onsite contact and services such as the library. Likewise, students mentioned onsite teaching with on-campus, in-person classes as the favored educational setup when suggesting new developments toward a new normal, such that teaching could provide more room for questions and interactions and thus be of higher quality. A few students preferred hybrid teaching, though they differed considerably regarding their specific interpretation. Some preferred practical courses and small group education taught onsite but larger lectures remaining online. Others preferred all courses taught both online and onsite, so students could choose how to attend. Finally, completely online teaching attracted some students, who listed advantages such as independence, decreased commuting time, and fewer concentration issues for those who struggled with ADHD or autism.

Notably, students emphasized asynchronous teaching as a promising future higher education practice. Uploading recorded lectures allows them to combine their studies with additional responsibilities (e.g., work), regulate their concentration (e.g., being able to pause the lecture), and prepare for exams (e.g., rewatching unclear parts). One student explicitly commented on the latter: “I have also noticed that the recordings of the lectures are very helpful when studying for exams.”

##### Social aspect of teaching

Students suggested how to enhance the social aspects of university education, mainly regarding onsite teaching. First, they wanted more interactive classes instead of one-sided lectures. Second, students preferred smaller class sizes to increase individual attention and better teacher interactions. Third, opportunities to connect, especially with fellow students, appeared essential. Non-content-related encounters outside the classroom could enable opportunities to get to know others. As a student explained, “Having lunch together and talking about issues […] is for me the thing I miss most during this period (talking about general issues in life with people you barely know).” Fourth, students accentuated social cohesion in education in general, for instance, through group assignments to feel more connected to their educational environment. One student described this need for community in the following way: “Onsite tutorials are definitely a great idea—get to see people, go to campus, feel like a part of it.”

## Discussion

This study investigated student well-being one year into the COVID-19 pandemic, from two perspectives: using the BPN to identify contextual factors, as well as an exploratory approach to examine students’ pandemic experiences in terms of their interactions and ideas for an educational new normal. To this end, we used a mixed-method approach to gather both qualitative and quantitative data. Ultimately, our results suggest three main findings. First, student well-being was compromised during the pandemic. Second, satisfaction and frustration of autonomy, competence, and relatedness needs related to student well-being in the expected directions, though the quantitative and qualitative results regarding relatedness produce some discrepancies. Third, university students stressed onsite education, recorded lectures, and the social aspects of student life as essential for the future ALE.

### Student well-being during COVID-19

Our findings hint at a relatively low level of student well-being one year into the pandemic. Compared with other studies using the WHO-5 scale to assess students’ well-being before the pandemic (e.g., Alessandri et al., 2020; Helou et al., 2019), students in our sample scored lower. Likewise, our positive and negative affect results indicate lower emotional well-being than the scores originally found when the scale was introduced (Watson et al., 1988). We see similar results when comparing our findings with other investigations of student well-being, resilience, and life satisfaction during the pandemic (e.g., Besser et al., 2020; Dopmeijer et al., 2021). This low level of well-being is concerning; it has been linked to students’ academic performance and adaptability to ALE changes (Besser et al., 2020; Stanton et al., 2016).

Although our initial research aims did not focus on relevant subgroups, we found some students to be more at risk for poor well-being than others. In particular, being a female student was related to more frequent negative affect, and being an international or STEM student was associated with lower well-being compared to Dutch students and students from non-STEM faculties, respectively. Our insight surrounding gender might be valuable, especially considering that they only differed regarding negative affect. These findings are in line with previous research that have found women to experience more frequent negative affect, as demonstrated with a Dutch sample when validating the Dutch PANAS (Engelen et al., 2006) as well as in STEM students when tutoring (Dumitru et al., 2022).

Our qualitative results echo comments particularly from first year and international students who reported struggling when interacting at the university. These findings also resonate with Dopmeijer et al.’s (2021) study, which showed that international students in particular report lower life satisfaction and well-being during the pandemic. These findings contrast pre-pandemic times, when no differences in well-being appeared between international and domestic students (Schofield et al., 2016). In contrast, first-year students repeatedly reported more compromised well-being than experienced students even before the pandemic (e.g., Bewick et al., 2010). This difference might stem from struggling with social belonging: Whereas first-year students had no pre-existing social network at the university even before the pandemic, some international students lost theirs because they returned home as soon as the pandemic hit. Regarding the differences among faculties, we suspect that the number of onsite contact hours may explain this variance; students stated that contact hours positively influenced how they perceived ALE interactions. Contact hours were limited at the STEM faculty, while most of the teaching remained onsite at the medical faculty. The size of the faculty also could account for a positive impact on well-being; for example, students from the comparably small spatial science faculty reported fewer negative emotions. Thus, studies that investigate how at-risk groups experienced the pandemic (Raaper et al., 2021) are particularly relevant for a fine-grained understanding of student well-being (Bond et al., 2021).

### Students’ relatedness within the ALE

The present study found that BPN satisfaction and frustration in the ALE predicted student well-being during COVID-19, though autonomy (H1.a and H2.a) and competence (H1.b and H2.b) were stronger factors than relatedness (H1.c and H2.c). Due to educational changes to remote teaching, we expected relatedness to be compromised. However, our quantitative and qualitative findings conflicted: Relatedness demonstrated the weakest relationship with well-being and the highest satisfaction and lowest frustration scores. Yet, in students’ qualitative responses, relatedness represented the main aspect students addressed when asked about their interactions at the university. Although students reported interactions to be scarce and distant, they emphasized the relevance of their sense of belonging to teachers and fellow students. Among their suggestions for future teaching, they often called for efforts to promote social cohesion.

The somewhat conflicting findings regarding relatedness seem surprising at first, given that previous research found that relatedness significantly influences student well-being (Sulea et al., 2015; Van den Broeck, 2008). However, other studies performed during the COVID-19 pandemic report similarly inconsistent findings. For example, Holzer et al. (2021) found that relatedness only marginally influenced students’ positive emotions. Competence, in contrast, had the strongest impact—a finding that corroborated our results. Researchers have offered various potential explanations for such counterintuitive findings. First, the understanding of relatedness may have shifted during the pandemic (Holzer et al., 2021). Social distancing altered the nature of connectedness throughout society, so assessing relatedness the same way as before may result in different interpretations. Second, we note a potential mismatch between faculty and student perceptions of higher education: Whereas faculty emphasize studying and identifying as a student, students prioritize personal relations (Naylor et al., 2021). Therefore, the university as an institution might need to promote autonomy and competence more than relatedness. Third, our study may have overestimated the relevance of relatedness, as the qualitative part of the questionnaire explicitly asked about students’ interactions, which may have primed students to talk about relatedness more than they would have if we had addressed their perceptions more generally.

### Implications for the future ALE

Building on students’ ideas for an educational new normal, we identify several implications for a future ALE: Students reported that they prefer onsite education, even if they accept hybrid teaching. Still, distinguishing between designed hybrid teaching and emergency remote teaching is necessary. The latter specifically refers to temporarily using online teaching tools due to an emergency situation, such as a pandemic (Hodges et al., 2020). In contrast, designed hybrid teaching entails more than mimicking onsite teaching online; the focus must be on designing an appropriate combination of online and offline teaching modes (e.g., flipped classroom, multimedia usage). Such an educational approach could enhance the quality of hybrid teaching, which might affect students’ satisfaction, motivation, and engagement (Ferrer et al., 2022).

Furthermore, students valued recorded lectures, which stand in line with prior research (Nordmann & McGeorge, 2018). However, a systematic review on teaching modes during COVID-19 revealed that teachers used synchronous online tools most often, which mimic face-to-face teaching (Bond et al., 2021). Asynchronous teaching enables students to study according to their personal learning styles (Ferrer et al., 2022). This freedom may increase their autonomy, which relates to student well-being. However, when students decide to watch lectures at their preferred time slot, relatedness in the ALE may be compromised. Therefore, we suggest further investigation of the ranking of the three BPNs in relation to student well-being, including comparisons with students’ wishes.

Finally, disrupted interactions at the university require focusing on social aspects in the ALE (Hagemeier & Dowling-McClay, 2020). Students suggested that interacting more frequently inside and outside class is valuable. Prior literature has emphasized the importance of the student–faculty relationship for student well-being as well, highlighting quantity, quality, and openness (Tormey, 2021; Trolian et al., 2020). Likewise, Giusti et al. (2021) stress that academic performance decreases if students switch from studying at a “social” university library to studying at home. Consequently, the Dutch Educational Council, as well as students and faculty themselves, express concerns about the lack of social character when teaching online (Eringfeld, 2021; ScienceGuide, 2022). Experts and students argue that learning happens through “real experiences, human interaction and physical expression,” which cannot be substituted but only complemented by technology (Abdrasheva et al., 2022, p. 11). Therefore, the future university must rebuild its social community character to prevent social disconnection (Hagemeier & Dowling-McClay, 2020).

### Limitations and further research

Despite this study’s strengths, there are several limitations that should be considered. First, our findings are context- and time-specific. Although the COVID-19 pandemic affected the entire world, countries handled the situation differently, implementing different measures at different times. Therefore, our findings must be interpreted in the specific context of the Netherlands in spring 2021. Moreover, we focused solely on one university and mainly STEM students, which may compromise the generalizability of the findings. Second, as we employed a cross-sectional design, our findings do not imply any causal relation. Third, we focused on students’ general and emotional well-being; we also acknowledge that well-being encompasses additional sub-facets that we did not investigate.

Considering these limitations and our discussion, continuing research should attend to specific at-risk groups, even after the pandemic has subsided. Researchers might also focus more specifically on relatedness and consider revising existing scales to fit to a (post-)pandemic context. Longitudinal designs also might explain the actual predictive power of factors within the ALE on student well-being.

## Conclusion

The present study aimed to investigate student well-being 1 year into the COVID-19 pandemic and which ALE factors contributed to it. Our findings highlight the importance of the BPN, particularly relatedness as a somewhat counterintuitive yet crucial aspect of interacting at the university. Moreover, the students in our sample provided various suggestions regarding a hybrid ALE and improved social cohesion. These findings may help initiatives shape the post-pandemic ALE and steer universities toward a healthier academic system that can thrive as a whole.

## Supplementary Information

Below is the link to the electronic supplementary material.

Supplementary file1 (PDF 1332 KB)

Supplementary file2 (PDF 614 KB)

Supplementary file3 (XLSX 58 KB)

Supplementary file4 (PDF 448 KB)

## Data Availability

A pre-print is already published and can be accessed at https://osf.io/5e96w. The data and supplementary material is openly available at dataverse.nl/dataset.xhtml?persistentId=doi:10.34894/WLTWCQ.

## Footnotes

1 We acknowledge the broadness of the term “well-being”; to keep the scope of this study manageable, we define it as the interplay of life satisfaction and the presence of positive and the absence of negative emotions (Beiser, 1974). For a more in-depth elaboration on well-being as a multifaceted construct, see Kiltz et al. (2020).

2 In contrast to the hypotheses, the RQs are not explicitly mentioned within our pre-registration and, thus, are explorative in nature. Moreover, the current study focuses solely on H2, as stated within the pre-registration.

3 The ethical request was issued under research plan number TED-2021-S-0015.

4 The pre-registration can be found at https://osf.io/5e96w.

5 In the Netherlands, students can follow a one-year pre-master track to switch from applied university to university.

6 The supplementary information I encompasses scale reliabilities, factor analysis of the Basic Psychological Need Satisfaction and Frustration Scale, assumption tests, analyses of potential confounding variables, and multiple regression models including control variables, a detailed description of the qualitative analysis; the supplementary information II includes the questionnaire; the supplementary information III a codebook with extended qualitative analyses, including frequencies and example quotes; and the supplementary information IV encompasses the used syntax.
