# Supplementary material for: Student well-being in times of COVID-19 in the Netherlands: basic psychological need satisfaction and frustration within the academic learning environment
Source: Eur J Psychol Educ. 2023 Mar 2:1–21. Online ahead of print. doi: 10.1007/s10212-023-00680-x (PMC9977641; doi:10.1007/s10212-023-00680-x)

Supplementary Material I

Scale reliabilities

The WHO-5 Well-being Index (World Health Organization, 1998) measures participants' general well-being on a 5-item scale. The respective 6-point Likert scale ranged from 1 ('at no time') to 6 ('all the time') and yielded a good level of internal consistency (Cronbach's α = .86). The 20-item Positive and Negative Affect Schedule (PANAS; Watson et al., 1988) ranged from 1 ('very slightly or not at all') to 5 ('extremely') and obtained good to excellent levels of internal consistency (Cronbach's α = .88 - .91). For the Basic Psychological Need Satisfaction and Frustration Scale (BPNSNF; Chen et al., 2015) we changed the items in so much that satisfaction and frustration of autonomy (e.g. 'I feel that my decisions reflect what I really want'), competence (e.g. 'I feel confident that I can do things well'), and relatedness (e.g. 'I feel that the people care about me') were assessed in relation to students' learning environment. Again, we asked participants to rate how they agreed to the statements over the two preceding weeks on a 5-point Likert scale ranging from 1 ('not at all true') to 5 ('totally true').

Factor analysis BPNSNF scale

We calculated an exploratory factor analysis fixed to the three factors originally defined and using maximum likelihood rotation to check the proposed factors for the BPNSNF scale. The results mainly supported the three-factor solution (with 30.6, 44.3, and 50.6% explained cumulative variance with each added factor). On an item level, only one item that was originally associated with relatedness ('I have the impression that people I spend time with dislike me.') loaded on the competence factor; otherwise, all items loaded on the respective predefined factors (see Table S1).

Assumption Testing

Herein, we report the assumption testing for each measure used for the main analysis within the paper. As correlational analyses require interval-scaled measures to be normally distributed, we tested these variables for normality using the Shapiro Wilk test (see Table S2). Unfortunately, all measures differed significantly from a normal distribution, hinting towards potential issues with the subsequent analyses. However, Field (2017) states that in larger samples, normality is of lesser interest, provided that the histograms show an approximation of a normal distribution. Considering the visual histograms (see Figure S1), we concluded that negative affect, relatedness satisfaction and frustration, as well as times of on-campus teaching violate the assumptions for Pearson correlation as the histograms were skewed. In this case, Spearman correlations are preferable. Therefore, the correlations comprising those variables display Spearman correlations instead of Pearson correlations (see Table 2 in the paper).

For the multiple regressions, we checked for the variance inflation factor (VIF) to check for meaningful serial correlations, as we expected the need satisfaction and frustration

measures to correlate significantly. However, the VIF for the measures ranged from 1.06 to 3.12 and, hence, indicated no issues in this regard. Additionally, the Durbin-Watson test indicates potential serial correlation between error terms. According to Field (2017), values between 1 and 3 or no cause for concern. In our data, the three multiple regressions calculated for the three outcome variables show values between 1.92 for negative affect and 2.11 for the WHO-5 measure. Therefore, we assumed that there is no serial correlation between the error terms within our data. Secondly, multiple regressions require homoskedasticity, which is assumed if the scatterplots show an arbitrary pattern when comparing the residuals. Moreover, errors should be normally distributed, which can be seen when comparing expected and observed values visually. Looking at Figure S2, both assumptions are met for all three multiple regression analyses. Lastly, the correlations amongst predictors may lead to problems with multicollinearity, which may, in turn, increase standard errors and β coefficients. As the predictors are partly intercorrelated in our study (see Table 2 in the paper), this could be a reason for concern. Consequently, it remains unclear which predictor accounts for how much variation within our regression model. Considering the tolerance values, however, values below .02 indicate that the regression may be biased (Field, 2017). However, that is not the case for any of the variables (see Table S2).

Identifying Control Variables

As preparation analysis for the multiple regression, we tested potential control variables for significant differences on one of the three well-being measures. Potential control variables included students' age, gender and whether they were in their bachelor's or master's, were international or Dutch, were the first in their family to enrol for higher education, were living alone or not, and at which faculty they studied. Except for the latter, all variables were either interval-scaled or dichotomous. Therefore, we first correlated these variables with both the well-being and need measures, generally Pearson correlations and only Spearman correlations only for the variables that were skewed (see Figure S1 & Table S3). As whether students were studying in their master or bachelor was the only variable not significantly correlating with either well-being or need measure, all other variables have to be considered as potential control variables for the successive multiple regression analysis.

For students' gender and study phase, we decided to exclude students identifying as 'other' and students following a pre-master programme, respectively. Those categories were too small to be statistically comparable to the other more prominent categories. However, we do want to provide the descriptives regarding these variables to show in which regard these students differed from the others (see Table S4). Based on these descriptives, students identifying as 'other' instead of as male or female reported lower well-being and need satisfaction, and higher need frustration at the same time. Pre-master students, however, did not differ from bachelor or master students.

As which faculty the students studied at was nominal-scaled, we opted for an ANOVA analysis to detect potentially meaningful difference between the different faculties. For the

analysis, the assumption of equal covariances (Box's test; p = .875) and homogeneity (Levene's test; p = .023 - .876) were met. Moreover, Pillai's Trace of .031 indicated that 31% of the variance within the well-being and need measures stem from the faculty affiliation. Likewise, the test of between-subject effects were significant for every well-being and need measure, except autonomy frustration (see Table S5). Therefore, we assumed that faculty affiliation should indeed be included as potential control variable. In more detail, multiple comparisons illustrate where exactly which faculties differ more clearly as post-hoc tests (see Table S6). As depicted in Table S6, all significant differences were between either the medical or the science and engineering faculty and the remaining faculties. Interestingly, the medical faculty differed from the remaining faculties solely on positive well-being and need satisfaction measures, with the exception of significant differences between the medical and science and engineering faculty. In contrast, the science and engineering faculty mainly differed significantly from the remaining faculties on negative measures, such as negative affect and need frustration. That is why we decided to include two dummy variables as control variables: One contrasting the medical faculty, the other contrasting the science and engineering faculty against the remaining faculties.

Consequently, the following potential control variables significantly related to either students' well-being or need satisfaction and frustration: which gender students identified with, how old they were, whether they lived alone or not, whether they were Dutch or international, whether they were the first ones in their family to attend university, and which faculty they studied at.

Multiple Regressions Including Control Variables

As explained in the main manuscript, we calculated a multiple regression with the well-being measures as outcomes and need satisfaction as predictors, using a two model analysis. The first model included solely the control variables as predictors, the second one the need satisfaction and frustration measures as well. In the manuscript, a shorter table is depicted, with certain information missing due to clarity. Therefore, we decided to include the extensive table in the supplementary material (Table S7).

Qualitative analysis

The qualitative analysis (content analysis; Grbich, 2012; Green & Thorogood, 2004) consisted of the following steps:

- Two coders read about 10% of the data to familiarise themselves with the data and start coding.
- Both coders labelled meaningful content of the answers. Based on these labels, they created a codebook for each of the three open-ended questions after a shared discussion meeting.

- Using these adjusted codebooks, both coders simultaneously coded the remaining 90% of the data. In an additional discussion meeting, they resolved discrepancies to assure understandability of the codes.
- Three coders categorised the codes into common themes.
- The resulting three final codebooks served as the basis for a final coding round executed one coder for 35% of the data, by another coder for 10% of the data, and by two other coders for the remaining data. Including independent coders yet unfamiliar with the codes helped to assure reliability.
- In a final meeting, two authors discussed complex cases and adjusted the codebook accordingly. For instance, ‘compatible with work’ became ‘compatible with life’ as it seemed more appropriate.
- Finally, one author finalised the content analysis creating a table for each code with a description of the word-level (descriptive) and co-occurring codes (interpretative).

References

Chen, B., Vansteenkiste, M., Beyers, W., Boone, L., Deci, E. L., der Kaap-Deeder, V., Duriez, B., Lens, W., Matos, L., Mouratidis, A., Ryan, R. M., Sheldon, K. M., Soenens, B., Van Petegem, S., & Verstuyf, J. (2015). Basic psychological need satisfaction, need frustration, and need strength across four cultures. Motivation and emotion, 39(2), 216-236. https://doi.org/10.1007/s11031-014-9450-1

Field, A. (2017). Discovering Statistics Using IBM SPSS (5th ed.). SAGE.

Grbich, C. (2012). *Qualitative data analysis: An introduction*. Sage.

Green, J., & Thorogood, N. (2004). Analysing qualitative data. In D. Silverman (Ed.), *Qualitative Methods for Health Research* (pp. 173-200). Sage.

Watson, D., Clark, L. A., & Tellegen, A. (1988). Development and validation of brief measures of positive and negative affect: the PANAS scales. Journal of Personality and Social Psychology, 54(6), 1063–1070. https://doi.org/10.1051/epjconf/201714006017

World Health Organization. (1998). WHO-Five Well-Being Index. Retrieved August 5, 2019, from https://www.psykiatri-regionh.dk/who-5/Documents/WHO5_English.pdf

Tables

Table S1.
Results for the rotated exploratory factor analysis

	Factor 1 (competence)	Factor 2 (relatedness)	Factor 3 (autonomy)
I feel insecure about my abilities. (c)	-.86	.06	-.06
I feel capable at what I do. (c)	.83	-.03	-.06
I feel confident that I can do things well. (c)	.82	.04	.01
I have serious doubts about whether I can do things well. (c)	-.81	.07	.07
I feel disappointed with many of my performances. (c)	-.76	-.05	-.00
I feel like a failure because of the mistakes I make. (c)	-.73	-.05	-.01
I feel I can successfully complete difficult tasks. (c)	.62	-.05	-.06
I feel competent to achieve my goals. (c)	.62	.08	-.14
I have the impression that people I spend time with dislike me. (r)	-.38	-.33	-.08
I feel close and connected with other people who are important to me. (r)	-.04	.90	.04
I feel connected with people who care for me, and for whom I care. (r)	-.11	.88	-.10
I experience a warm feeling with the people I spend time with. (r)	-.08	.82	-.01
I feel that the people care about me. (r)	-.03	.71	-.02
I feel the relationships I have are just superficial. (r)	-.06	-.62	-.05
I feel that people who are important to me are cold and distant towards me. (r)	-.14	-.42	.06
I feel excluded from the group of people. (r)	-.19	-.33	.10
Most of the things I do feel like 'I have to'. (a)	.04	.03	.76
My daily activities feel like a chain of obligations. (a)	-.02	-.02	.73
I feel forced to do many things I wouldn't choose to do. (a)	.03	.01	.73
I feel pressured to do too many things. (a)	.04	.05	.68
I feel I have been doing what really interests me. (a)	.14	.05	-.52
I feel a sense of choice and freedom in the things I undertake. (a)	.04	.10	-.49
I feel that my decisions reflect what I really want. (a)	.24	.12	-.33
I feel my choices express who I really am. (a)	.28	.09	-.32

Note. Extraction method: maximum likelihood; rotation method: oblimin with kaiser normalization; c = affiliated with original competence sub-factor, r = affiliated with original relatedness sub-factor, a = affiliated with original autonomy sub-factor

Table S2.

Results for Shapiro-Wilk tests and tolerance values of the three multiple regressions

	Shapiro-Wilk-test *W* (df)	Tolerance
WHO-5[a]	.984 (613)**	–
Positive affect[a]	.992 (613)**	–
Negative affect[a]	.968 (613)**	–
Autonomy (S)	.983 (613)**	.496
Autonomy (F)	.984 (613)**	.638
Competence (S)	.979 (613)**	.320
Competence (F)	.970 (613)**	.327
Relatedness (S)	.967 (613)**	.578
Relatedness (F)	.949 (613)**	.501
Gender[b]	–	.947
Nationality[b]	–	.812
Living alone[b]	–	.884
First generation student[b]	–	.899
Age	.899 (613)**	.905
M affiliation[b]	–	.716
SE affiliation[b]	–	.741

Note. * $p < .05$, ** $p < .01$; S = satisfaction, F = frustration, M/SE affiliation = affiliation with the medical/science and engineering faculty; for multiple regression, only the model including the control variables is reported; [a]for outcome variables of the multiple regression, no tolerance values are reported, [b]dichotomous, nominal- and ordinal-scaled measures were not tested for normality

Table S3.

Correlations amongst the potential control variables and well-being as well as need measures

	1	2	3	4	5	6	7	8	9	10	11	12	13	14	15
1 WHO-5	1	–	–	–	–	–	–	–	–	–	–	–	–	–	–
2 Positive affect	.810**	1	–	–	–	–	–	–	–	–	–	–	–	–	–
3 Negative affect[a]	-.615**	-.490**	1	–	–	–	–	–	–	–	–	–	–	–	–
4 Autonomy satisfaction	.551**	.580**	-.362**	1	–	–	–	–	–	–	–	–	–	–	–
5 Autonomy frustration	-.514**	-.421**	.421**	-.558**	1	–	–	–	–	–	–	–	–	–	–
6 Competence satisfaction	.598**	.623**	-.502**	.599**	-.432**	1	–	–	–	–	–	–	–	–	–
7 Competence frustration	-.618**	-.596**	.626**	-.468**	.451**	-.777**	1	–	–	–	–	–	–	–	–
8 Relatedness satisfaction[a]	.462**	.478**	-306**	.398**	-.289**	.390**	-.383**	1	–	–	–	–	–	–	–
9 Relatedness frustration[a]	-.393**	-.370**	.400**	-.311**	.312**	-.364**	.495**	-.604**	1	–	–	–	–	–	–
10 Age[a]	-.077	-.067	.077	-.087*	.028	-.060	.050	-.022	-.053	1	–	–	–	–	–
11 Gender (woman 1, man 2)	-.012	.019	.173**	.018	.036	-.041	.085*	.070	.019	-.021	1	–	–	–	–
12 Bachelor (1) vs. Master (2)	.016	.031	.014	.020	-.029	.056	-.042	.017	-.070	.665**	.084*	1	–	–	–
13 International (1) vs. Dutch (2)	.166**	.123**	-.146**	-.005	-.054	.034	-.160**	.123**	-.195**	.027	-.007	.067	1	–	–
14 First generation (1) vs. not (2)	.031	-.009	.091*	.004	.010	-.020	.035	-.014	.041	-.055	.048	-.068	-.299**	1	–
15 Living alone (1) vs. not (2)	.104**	.082*	-.031	.058	-.017	.042	-.053	.074	-.073	.000	.025	-.142**	.201**	-.001	1

Note. * $p < .05$, ** $p < .01$; [a]Spearman correlation; (1) and (2) refer to the value the corresponding dichotomous option had assigned

Table S4.

Mean differences on well-being and need-measures regarding students' gender and study phase, with standard errors in brackets

	gender		
	male	female	other
WHO-5	9.8 (0.3)	9.7 (0.2)	6.7 (1.5)
Positive affect	25.9 (0.5)	26.2 (0.4)	22.8 (2.2)
Negative affect	22.0 (0.5)	24.9 (0.4)	29.4 (2.6)
Autonomy satisfaction	3.2 (0.1)	3.2 (0.0)	2.6 (0.3)
Autonomy frustration	3.2 (0.1)	3.3 (0.0)	3.8 (0.4)
Competence satisfaction	3.2 (0.1)	3.2 (0.0)	2.6 (0.4)
Competence frustration	2.8 (0.1)	2.9 (0.1)	3.5 (0.4)
Relatedness satisfaction	3.4 (0.1)	3.5 (0.1)	2.6 (0.2)
Relatedness frustration	2.1 (0.1)	2.2 (0.0)	3.0 (0.4)
	study phase		
	Bachelor	Pre-master	Master
WHO-5	9.6 (0.3)	10.1 (0.9)	9.8 (0.3)
Positive affect	25.8 (0.4)	26.8 (1.4)	26.3 (0.5)
Negative affect	23.8 (0.4)	24.1 (1.6)	24.1 (0.5)
Autonomy satisfaction	3.2 (0.0)	3.1 (0.2)	3.2 (0.1)
Autonomy frustration	3.3 (0.0)	3.5 (0.2)	3.2 (0.1)
Competence satisfaction	3.2 (0.0)	3.2 (0.2)	3.2 (0.1)
Competence frustration	2.9 (0.1)	2.9 (0.2)	2.8 (0.1)
Relatedness satisfaction	3.5 (0.1)	3.6 (0.2)	3.5 (0.1)
Relatedness frustration	2.2 (0.1)	2.2 (0.2)	2.1 (0.1)

Table S5.

Between-subject effects for the ANOVA regarding faculty affiliation on well-being and need measures

	df	*F*	*p*
WHO-5	5	5.38	.000
Positive affect	5	5.24	.000
Negative affect	5	3.828	.002
Autonomy satisfaction	5	3.836	.002
Autonomy frustration	5	.762	.577
Competence satisfaction	5	4.04	.001
Competence frustration	5	3.64	.003
Relatedness satisfaction	5	2.81	.016
Relatedness frustration	5	2.70	.020

Table S6.

Multiple Comparisons for the ANOVA regarding faculty affiliation on well-being and need measures

		*M*Δ(*SE*)								
		WHO-5	Positive affect	Negative affect	Autonomy satisfaction	Autonomy frustration	Competence satisfaction	Competence frustration	Relatedness satisfaction	Relatedness frustration
BS	M	-1.42(.752)	-2.86(1.14)*	.922(1.24)	-.290(.114)*	.213(.135)	-.303(.125)*	.272(.160)	-.111(.137)	-.108(.128)
	SS	.022(.780)	.684(1.18)	1.59(1.28)	.073(.118)	.141(.139)	-.068(.130)	.187(.166)	.137(.142)	-.134(.133)
	SE	1.23(.687)	.951(1.04)	-1.73(1.13)	.004(.104)	.144(.123)	.079(.114)	-.149(.146)	.199(.125)	-.321(.117)**
	other	.261(1.30)	-.105(1.97)	-1.54(2.13)	.081(.200)	-.051(.232)	-.125(.216)	.184(.276)	.404(.237)	.055(.221)
	> 2F	-.183(1.30)	-2.05(1.97)	-2.20(2.13)	-.099(.197)	.005(.232)	-.125(.216)	.003(.276)	.210(.237)	-.140(.221)
M	BS	1.42(.752)	2.86(1.14)*	-.922(1.24)	.290(.114)*	-.213(.135)	.303(.125)*	-.272(.160)	.111(.137)	.108(.128)
	SS	1.44(.637)*	3.55(.966)**	.670(1.05)	.363(.096)**	-.072(.114)	.235(.106)*	-.085(.135)	.247(.116)*	-.025(.108)
	SE	2.65(.518)**	3.81(.787)**	-2.65(851)**	.294(.078)**	-.069(.093)	.382(.086)**	-.421(.110)**	.310(.095)**	-.213(.088)*
	other	1.68(1.22)	2,76(1.85)	-2.46(2.00)	.371(.184)*	-.264(.218)	.178(.203)	-.089(.259)	.515(.222)*	.163(.207)
	> 2F	1.24(1.22)	.814(1.85)	-3.13(2.00)	.191(.184)	-.209(.218)	.178(.208)	-.269(.259)	.320(.222)	-.032(.207)
SS	BS	-.022(.780)	-.684(1.18)	-1.59(1.28)	-.073(.118)	-.141(.139)	.068(.130)	-.187(.166)	-.137(.142)	.134(.133)
	M	-1.44(.637)*	-3.55(.966)**	-.670(1.05)	-.363(.097)**	.072(.114)	-.235(.106)*	.085(.135)	-.247(.116)*	.025(.108)
	SE	1.21(.557)*	.267(.846)	-3.32(.915)**	.069(.084)	.003(.100)	.147(.093)	-.336(.118)**	.063(.102)	-.188(.095)*
	other	.240(1.24)	-.789(1.88)	-3.13(2.03)	.008(.187)	-.192(.221)	-.057(.206)	-.003(.262)	.268(.226)	.188(.210)
	> 2F	-.205(1.236)	-2.73(1.88)	-3.79(2.03)	-.172(.187)	-.137(.221)	-.057(.206)	-.184(.262)	.073(.226)	-.006(.210)
SE	BS	-1.23(.687)	-.951(1.04)	1.73(1.13)	-.004(.104)	-.144(.123)	-.079(.114)	.149(.146)	-.199(.125)	.321(.117)**
	M	-2.65(.518)**	3.81(.787)**	2.65(.851)**	-.294(.078)**	.069(.093)	-.382(.086)**	.421(.110)**	-.310(.095)**	.213(.088)*
	SS	-1.21(.557)*	-.267(.846)	3.32(.915)**	.069(.084)	-.003(.100)	-.147(.093)	.336(.118)**	-.063(.102)	.189(.095)**
	other	-.967(1.18)	1.06(1.79)	.196(1.94)	.077(.178)	-.196(.211)	-.204(.196)	.332(.250)	.205(.215)	.376(.201)
	> 2F	-1.41(1.18)	-3.00(1.79)	-.470(1.94)	-.103(.178)	-.140(.211)	-.204(.196)	.152(.250)	.010(.215)	.182(.201)
other	BS	-.261(1.30)	.105(1.97)	1.54(2.13)	-.08(.197)	.051(.232)	.1245(.216)	-.184(276)	-.404(.237)	-.055(.221)
	M	-1.68(1.22)	-2.76(1.85)	2.46(2.00)	-.371(.184)*	.264(.218)	-.178(.203)	.089(.259)	-.515(.222)*	-.163(.207)
	SS	-.240(1.24)	.789(1.88)	3.13(2.03)	-.008(.187)	.192(.221)	.057(.206)	.003(.262)	-.268(.226)	-.188(.210)
	SE	.967(1.18)	1.06(1.79)	-.196(1.94)	-.077(.178)	.195(.211)	.204(.196)	-.332(.250)	-.205(.215)	-.376(.201)
	> 2F	-.444(1.62)	-1.94(2.45)	-.667(2.65)	-.181(.244)	.056(.289)	.000(.269)	-.181(.343)	.194(.295)	.194(.275)
> 2F	BS	.183(1.30)	2.05(1.97)	2.20(2.13)	.099(.197)	-.005(.232)	.125(.216)	-.003(.276)	-.210(.237)	.140(.221)
	M	-1.24(1.22)	-.814(1.85)	3.13(2.00)	-.191(.184)	.209(.218)	-.178(.203)	.269(.259)	-.320(.222)	.032(.207)
	SS	.205(1.24)	2.73(1.88)	3.79(2.03)	.172(.187)	.137(.221)	.057(.206)	.184(.262)	-.073(.226)	.006(.210)

SE	1.41(1.18)	3.00(1.79)	.470(1.94)	.103(.178)	.140(.211)	.204(.196)	-.152(.250)	-.010(.215)	.194(.275)
other	.444(1.62)	1.94(2.45)	.667(2.65)	.181(.244)	-.056(.289)	.000(.269)	.181(.343)	.194(.295)	-.182(.201)

Note. * $p < .05$, ** $p < .01$; BS = behavioural and social sciences faculty, MF = medical faculty, SS = spatial sciences faculty, SE = science and engineering faculty, other = other faculties, > 2F = more than two faculties

Table S7.

Summary of the multiple regressions with WHO-5, positive and negative affect as the outcome variable, respectively, students' need satisfaction and frustration as predictors, and their sociodemographics as control variables.

	WHO-5					Positive Affect					Negative Affect				
	B	*SE B*	*β*	t	95% CI (*B*)	*B*	*SE B*	*β*	t	95% CI (*B*)	*B*	*SE B*	*β*	t	95% CI (*B*)
**Model 1**															
Gender	-.133	.419	-.013	.318	-.955 – .689	.204	.638	.013	.320	-1.05 – 1.46	2.77	.667	.168	4.15**	1.46 – 4.08
Nationality	1.89	.514	.158	3.67**	.875 – 2.90	2.43	.782	.135	3.11**	.895 – 3.97	-2.74	.822	-.143	-3.37**	-4.36 – -1.13
Living alone	.270	.526	.022	.514	-.762 – 1.30	.099	.804	.005	.124	-1.48 – 1.95	.928	.841	.047	1.10	-.723 – 2.58
First gen	.159	.437	.015	.363	-.699 – 1.02	.641	.667	.041	.960	-.670 – 1.95	.618	.697	.037	.886	-.752 – 1.99
Age	-.200	.083	-.100	-2.40*	-.364 – -.036	-.308	.127	-.101	-2.42*	-.557 – -.058	.399	.133	.124	3.00**	.138 – .661
M affiliation	.326	.138	.110	2.37*	.055 – .596	.701	.209	.156	3.35**	.289 – 1.11	-.180	.219	-.038	-.822	-.611 – .250
NS affiliation	-.162	.065	-.114	-2.49*	-.290 – -.034	-.095	.100	-.044	-.954	-.290 – .101	.312	.104	.136	2.99**	.107 – .516
Adjusted R^2	.062					.049					.074				
F	6.48					5.29					7.64				
**Model 2**															
Gender	.142	.302	.014	.469	-.451 – .735	.553	.464	.035	1.19	-.358 – 1.46	1.87	.521	.113	3.59**	.846 – 2.90
Nationality	1.23	.377	.104	3.27**	.493 – 1.98	1.803	.579	.100	3.11**	.665 – 2.94	-1.06	.654	-.055	-1.62	-2.35 – .225
Living alone	.319	.376	.026	.848	-.419 – 1.06	-.085	.580	-.005	-.147	-1.22 – 1.05	.731	.651	.037	1.12	-.548 – 2.01

First gen	.101	.312	.010	.324	-.512 – .714	.558	.481	.036	1.16	-.386 – 1.50	.765	.540	.046	1.42	-.295 – 1.83
Age	-.080	.060	-.040	-1.33	-.198 – .038	-.089	.093	-.029	-.955	-.271 – .094	.293	.104	.091	2.81**	.088 – .497
M affiliation	.040	.100	.014	.404	-.156 – .237	.216	.154	.048	1.41	-.085 – .518	.022	.173	.005	.125	-.318 – .361
NS affiliation	-.123	.047	-.086	-2.61**	-.216 – -.030	-.012	.073	-.006	-.165	-.155 – .131	.171	.082	.075	2.10*	.011 – .331
Autonomy (S)	1.15	.273	.171	4.21**	.613 – 1.68	2.54	.417	.251	6.08**	1.72 – 3.36	.099	.469	.009	.210	-.823 – 1.02
Autonomy (F)	-1.14	.205	-.198	-5.54**	-1.54 – -.734	-.415	.316	-.047	-1.31	-1.04 – .206	1.50	.355	.162	4.21**	.798 – 2.19
Competence (S)	.841	.310	.137	2.71**	.231 – 1.45	1.93	.478	.206	4.03**	.988 – 2.86	-.367	.537	-.037	-.683	-1.42 – .688
Competence (F)	-1.27	.241	-.264	-5.29**	-1.74 – -.799	-1.66	.370	-.227	-4.49**	-2.39 – -.934	3.42	.416	.442	8.01**	2.60 – 4.24
Relatedness (S)	.953	.209	.172	4.57**	.543 – 1.36	1.70	.321	.201	5.29**	1.07 – 2.33	-.085	.362	-.010	-.235	-.795 – .625
Relatedness (F)	.181	.245	.030	.739	-.300 – .661	.420	.376	.046	1.12	-.318 – 1.16	1.11	.424	.114	2.62**	.278 – 1.95
Adjusted R^2	.523					.508					.447				
F	50.26					46.38					37.42				

Note. *p < .05, **p < .01; S = Satisfaction, F = Frustration, First gen = first generation student, M affiliation = affiliation with medical faculty, NS affiliation = affiliation with natural sciences faculty; 95%CI = 95% CI for the *B*.

Figures

Figure S1.

Histogram for interval-scaled measures

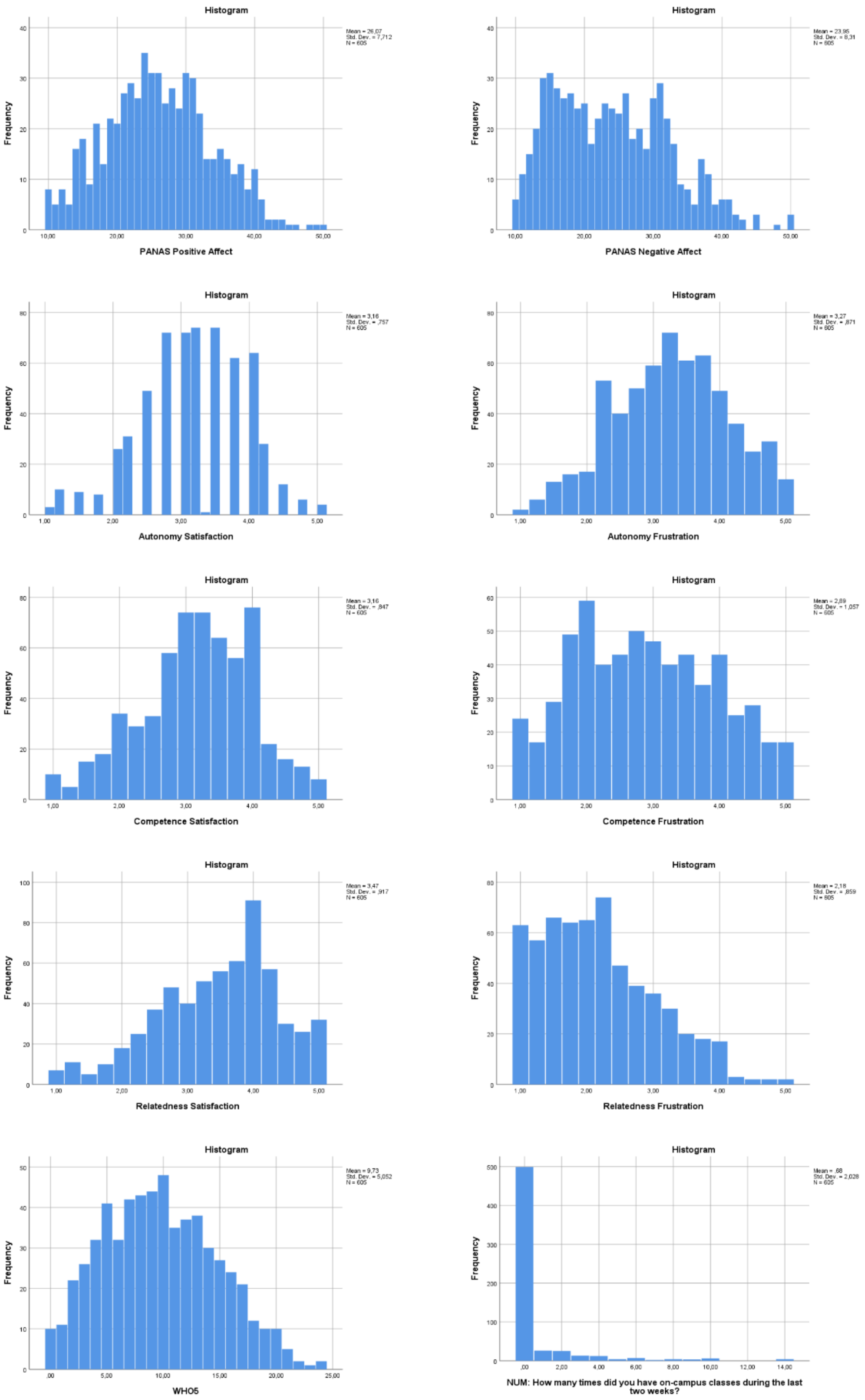

Figure S2.

(a) Scatterplot and (b) normal PP-plot of regression standardised residual for all three multiple regressions

(a) (b)

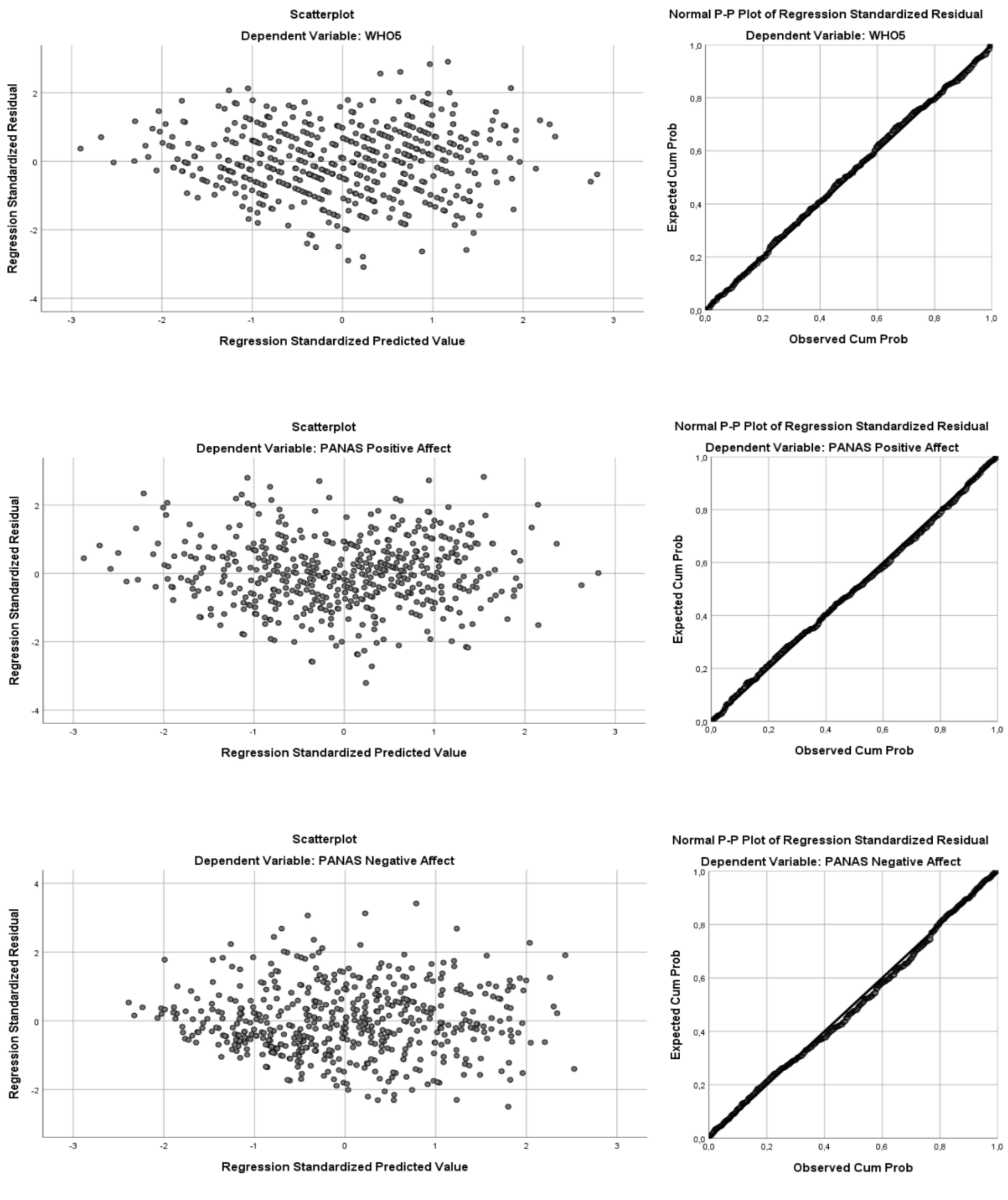

Supplement: Supplementary file 1 — Supplementary file1 (PDF 1332 KB) [file 10212_2023_680_MOESM1_ESM.pdf]
