# Supplementary material for: Student well-being in times of COVID-19 in the Netherlands: basic psychological need satisfaction and frustration within the academic learning environment
Source: Eur J Psychol Educ. 2023 Mar 2:1–21. Online ahead of print. doi: 10.1007/s10212-023-00680-x (PMC9977641; doi:10.1007/s10212-023-00680-x)
Supplement: Supplementary file 2 — Supplementary file2 (PDF 614 KB) [file 10212_2023_680_MOESM2_ESM.pdf]

Supplementary Material II

Survey Flow

Block: Introduction (1 Question)
Standard: Informed consent (1 Question)
Standard: Help and resources (1 Question)
Standard: Sociodemographics (7 Questions)
Standard: Study-related questions (15 Questions)
Standard: COVID-19-related (10 Questions)
Standard: Well-being (2 Questions)
Standard: Individual predictors I (1 Question)
Standard: Halftime! (2 Questions)
Standard: Individual Predictors II (4 Questions)
Standard: Learning environment-related factors (6 Questions)
Standard: End of Questionnaire (4 Questions)

Start of Block: Introduction

Q39 Thank you for participating in this survey! In doing this, you will not only provide information to your respective faculty to give them the opportunity to act upon it, but also support a PhD project focused on student well-being and, hence, support the research field surrounding the topic.
First of all, we want to give you some practical information about the survey.

Do I have to participate in this research? Participation in the research is voluntary. If you decide not to participate, you do not need to explain why, and there will be no negative consequences for you. You can terminate participation at any time without giving an explanation and without consequences by simply closing your internet browser. If you decide to withdraw your consent, the data that will have been collected up to that point may be used for research purposes.

Why do we conduct this research? Our aim is to investigate the current state of well-being of [...] students during the COVID-19 pandemic. We are also looking into individual factors and factors within your learning environment to see how these influence your well-being. All researchers involved are affiliated to the [...] or the [...].

What do we ask of you during the research? You will be asked to answer an online questionnaire. It is important to clarify that we will ask about your nationality, your age, and which year you are in your studies. You can choose the option “Prefer not to say” if you do not want to answer these questions. Furthermore, you may experience some discomfort upon answering certain questions, but all questions are completely voluntary. As a result of reflecting on your well-being and study experiences in the surveys, your self-awareness and psychological awareness may change. Moreover, we want to point out that there are no right or wrong answers and we highly appreciate you answering as honestly and spontaneously as possible.

How will we treat your data? Each respondent will be assigned an identification number so the researchers will not be able to identify you as an individual. Furthermore, all data will be treated according to the guidelines of the [...] Faculty. All of your responses will processed and stored confidentially. Only authorized researchers that are involved in this study will have access to information you provide in the survey. Information will be reported in aggregate form (i.e., summarized at the total sample level) in a way that individual participants cannot be identified.
After data analysis, we plan on publishing the data openly. However, this does not mean that your data can be traced back to you, as we will remove all sociodemographic identifiers. Only upon request, professional researchers who are interested in reusing the anonymized data for future studies will be granted access to these additional information.

What else do you need to know? You may always ask questions about the research: now, during the research, and after the end of the research. If you have questions about this study, please contact [...] or [...].

Do you have questions or concerns regarding your rights as a research participant? For this you may also contact the Ethics Committee [...]. Do you have questions or concerns regarding your privacy, or regarding the handling of your personal data? For this you may also contact the Data Protection Officer of the [...].

As a research participant you have the right to make a copy of this research information.

End of Block: Introduction

Start of Block: Informed consent

Q37 I hereby declare that

- I am 16 years or older.
- I have been informed about this research satisfactorily.
- I have read the information and understand what is expected from me and understood the information properly.
- I have had the opportunity to ask questions about the research. I know that my participation is voluntary and I have been informed about my rights.
- I also know that I can end my participation at any moment, without explaining why. I understand how my data will be processed and protected. I understand the text above and I agree with the participation in this research.

○ I agree (1)

○ I disagree (2)

Skip To: End of Survey If Q37 = 2

End of Block: Informed consent

Start of Block: Help and resources

Q55 Before we start with the questionnaire, we want to acknowledge the strain on your well-being during the last year. A pandemic such as the current can be hard and challenging. We know that asking you for your perception of the last couple of months may arise negative emotions and memories and don't want to leave you alone with this. If you have lost a loved one during the pandemic or experienced anxiety, distress, or depressive moods in the past months, you can turn to the [...] for short-term (online) counselling and psychological help. Additionally, the [...] provides a short overview with tips and tricks to remain mentally healthy during the COVID-19 pandemic. Moreover, we will provide you with an additional PDF on how to maintain your psychological well-being despite the current circumstances at the end of this questionnaire as free download.

End of Block: Help and resources

Start of Block: Sociodemographics

Q3 First, we would like to pose some questions about yourself and your general living situation.

Q4 What is your age?

- ○ My age is: (1) ____

- ○ Prefer not to say (2)

Q5 What is your current gender identity?

- ○ Male (1)

- ○ Female (2)

- ○ Other (3)

- ○ Prefer not to say (4)

Q6 Are you an international student?
(i.e. a citizen of another country studying in the Netherlands)

- ○ Yes (1)

- ○ No (2)

- ○ Prefer not to say (3)

Q7 What is your nationality?
(if you have 2 or more, please indicate the one you feel most connected to)

- ○ Dutch (1)
- ○ Other European (2)
- ○ Non-European (3)
- ○ Prefer not to say (4)

Q8 With whom are you currently living?
(multiple answers possible)

- ☐ Alone (1)
- ☐ Housemate(s) (2)
- ☐ Partner (3)
- ☐ Parent(s)/sibling(s) (4)
- ☐ With my child(ren) (5)
- ☐ Other, namely: (6) ____
- ☐ Prefer not to say (7)

Q9 Are you in a relationship?

○ No (1)

○ Yes, for more than a year (2)

○ Yes, for less than a year (3)

○ Prefer not to say (4)

End of Block: Sociodemographics

Start of Block: Study-related questions

Q8 Further, we would like to know a bit more about your studies and how these may have been impacted by the COVID-19 pandemic.

Q9 Are you a Bachelor or a Master student?

○ Bachelor student (1)

○ Pre-master student (2)

○ Master student (3)

○ Prefer not to say (4)

Q10 Which year of your current studies are you in?

- ○ 1st year (1)
- ○ 2nd year (2)
- ○ 3rd year (3)
- ○ 4th year and beyond (4)
- ○ Prefer not to say (5)

Q11 What faculty do you belong to?
(multiple answers possible)

- [ ] [Faculty 1] (1)
- [ ] [Faculty 2] (2)
- [ ] [Faculty 3] (3)
- [ ] [Faculty 4] (4)
- [ ] [Faculty 5] (5)
- [ ] [Faculty 6] (6)
- [ ] [Faculty 7] (7)
- [ ] [Faculty 8] (8)
- [ ] [Faculty 9] (9)
- [ ] [Faculty 10] (10)
- [ ] [Faculty 11] (11)
- [ ] Prefer not to say (12)

Q12 Are you the first in your family to enroll in university education?

- [ ] Yes (1)
- [ ] No, (one of) my (great) parents enrolled in university education (2)
- [ ] No, (one of) my older siblings is or was enrolled in university education (3)
- [ ] Prefer not to say (4)

Q13 Do you have an academic delay due to COVID-19?

- Yes (1)
- Likely (2)
- Likely not (3)
- No (4)
- Prefer not to say (5)

Display This Question:

If Q13 = 1

And Q13 = 2

Q14 How long will your academic delay presumable be?

- 1 semester or less (1)
- 2 semesters (2)
- More than 2 semesters (3)
- Prefer not to say (4)

Display This Question:

If Q13 = 1

And Q13 = 2

Q15 What is the reason for your academic delay?

____

Q16 How do you feel the COVID-19 pandemic has changed your study-related workload, as compared to before COVID-19?

- ○ Higher (1)
- ○ More or less the same (2)
- ○ Lower (3)
- ○ I have never studied before COVID-19 (4)
- ○ Prefer not to say (5)

Q17 How many times did you have on-campus classes during the last two weeks?

____

Q18 Do you have access to an adequate study space at home?

- ○ Yes (1)
- ○ No, but I can go to a study space at the university (4)
- ○ No (2)
- ○ Prefer not to say (3)

Display This Question:

If Q18 = 2

Q19 You don't have an adequate study space at home according to the previous question, what is missing?

____

Q20 Do you have access to adequate equipment for studying (including internet, headset, etc)?

- Yes (1)
- No (2)
- Prefer not to say (3)

Display This Question:

If Q20 = 2

Q21 What is missing in equipment for studying?

____

Q22 As you might know, the university offers (short-time) psychological counselling at the [...]. If you were experiencing any difficulties due to the pandemic (because of COVID-19 itself, but also because of the restrictions coming along with it), would you consider making use of this offer?

- Definitely would go (1)
- Probably would go (2)
- Might or might not go (3)
- Probably would not go (4)
- Definitely would not go (5)
- Prefer not to say (6)

End of Block: Study-related questions

Start of Block: COVID-19-related

Q23 To better understand your pandemic-related situation, we would like to know how you experienced the COVID-19 pandemic. We know that these questions can be quite personal and want to remind you that this questionnaire is completely anonymised.

Q24 Have you been diagnosed with COVID-19?

- Yes (1)
- No (2)
- No, but I think I've had it nonetheless (3)
- Prefer not to say (4)

Q25 Has anybody near you (housemate, near friend, family member) been diagnosed with COVID-19?

- Yes (1)
- No (2)
- Prefer not to say (3)

Display This Question:

If Q25 = 1

Q26 Has it been a serious or even fatal course of disease?

- Yes (1)
- No (2)
- Prefer not to say (3)

Q27 Have you been quarantined due to symptoms, contact to COVID-19 positive individuals, or travelling abroad?

- Yes, more than once (1)
- Yes, once (2)
- No (3)
- Prefer not to say (4)

Q28 Do you belong to an at-risk group (e.g. overweight or underlying health conditions)?

- No (1)
- Yes, by definition (2)
- Yes, not by definition, but I do feel vulnerable to COVID-19 nonetheless (3)
- Prefer not to say (4)

Q29 How much time in total did you spend actively looking for COVID-19-related news daily during the last seven days?

- I did not actively search for COVID-19 related news (1)
- Less than an hour (2)
- Between one and two hours (3)
- More than two hours (4)
- Prefer not to say (5)

Q30 During the last seven days, how much time per day did you spend outside on average?

- Less than 30 minutes per day (1)
- Between 30 minutes and an hour per day (2)
- More than an hour per day (3)
- Prefer not to say (4)

Q31 How anxious did you feel due to the pandemic during the last two weeks on a scale from 0 (not anxious) to 10 (extremely anxious)?

	Not anxious					Medium					Extremely anxious
	0	1	2	3	4	5	6	7	8	9	10
1 ()											

Q32 How was your concentration on your studies during the last two weeks on a scale from 0 (not concentrated) to 10 (extremely concentrated)?

	Not concentrated					Medium					Extremely concentrated
	0	1	2	3	4	5	6	7	8	9	10
1 ()											

End of Block: COVID-19-related

Start of Block: Well-being

Q34 The following statements concern how you have been feeling lately. Remember, virtually everyone experiences phases in their lives during which they feel down, sad, or anxious, which may be even more frequently or severe during a stressful period such as the current. Therefore, please indicate for each of the five statements which is closest to how you have been feeling over the last two weeks.
Over the last two weeks,

	At no time (1)	Some of the time (2)	Less than half of the time (3)	More than half of the time (4)	Most of the time (5)	All the time (6)
I have felt cheerful and in good spirits (1)	o	o	o	o	o	o
I have felt calm and relaxed (2)	o	o	o	o	o	o
I have felt active and vigorous (3)	o	o	o	o	o	o
I woke up feeling fresh and rested (4)	o	o	o	o	o	o
My daily life has been filled with things that interests me (5)	o	o	o	o	o	o

Q35 Below you see a number of words that describe different feelings and emotions. Please read each word and indicate to what extent you felt the respective emotion during the past two weeks on a scale ranging from 'very slightly or not at all' to 'extremely'.

	Very slightly or not at all (1)	A little (2)	Moderately (3)	Quite a bit (4)	Extremely (5)
Enthusiastic (1)	○	○	○	○	○
Interested (2)	○	○	○	○	○
Determined (3)	○	○	○	○	○
Excited (4)	○	○	○	○	○
Inspired (5)	○	○	○	○	○
Alert (6)	○	○	○	○	○
Active (7)	○	○	○	○	○
Strong (8)	○	○	○	○	○
Proud (9)	○	○	○	○	○
Attentive (10)	○	○	○	○	○
Scared (11)	○	○	○	○	○
Afraid (12)	○	○	○	○	○
Upset (13)	○	○	○	○	○
Distressed (14)	○	○	○	○	○
Jittery (15)	○	○	○	○	○

Nervous (16)	o	o	o	o	o
Ashamed (17)	o	o	o	o	o
Guilty (18)	o	o	o	o	o
Irritable (19)	o	o	o	o	o
Hostile (20)	o	o	o	o	o

End of Block: Well-being

Start of Block: Individual predictors I

Q36 In this part, we would like to ask you some questions about your self-compassion.

Please read each statement carefully before answering and indicate how often you behave in the stated manner, using a scale ranging from 'almost never' to 'almost always'. These questions aim at how you usually cope with adversities, so do not exclusively think about how you cope with the pandemic, but also with prior difficulties.

	Almost never (1)	Sometimes (2)	About half the time (3)	Most of the time (4)	Almost always (5)
When I fail at something important to me, I become consumed by feelings of inadequacy. (1)	○	○	○	○	○
I try to be understanding and patient towards those aspects of my personality I don't like. (2)	○	○	○	○	○
When something painful happens, I try to take a balanced view of the situation. (3)	○	○	○	○	○
When I'm feeling down, I tend to feel like most other people are probably happier than I am. (4)	○	○	○	○	○
I try to see my failings as part of the human condition. (5)	○	○	○	○	○

When I'm going through a very hard time, I give myself the caring and tenderness I need. (6)	○	○	○	○	○
When something upsets me, I try to keep my emotions in balance. (7)	○	○	○	○	○
When I fail at something that's important to me, I tend to feel alone in my failure. (8)	○	○	○	○	○
When I'm feeling down, I tend to obsess and fixate on everything that's wrong. (9)	○	○	○	○	○
When I feel inadequate in some way, I try to remind myself that feelings of inadequacy are shared by most people. (10)	○	○	○	○	○

I’m disapproving and judgmental about my own flaws and inadequacies. (11)	○	○	○	○	○
I’m intolerant and impatient towards those aspects of my personality I don’t like. (12)	○	○	○	○	○

End of Block: Individual predictors I

Start of Block: Halftime!

Q42 Great! You now answered half of the questionnaire!

End of Block: Halftime!

Start of Block: Individual Predictors II

Q45 Furthermore, we would like to know how the current pandemic reflects on your concentration and attention capacities. Please indicate as honestly as possible the extent to which the following statements are true for you, using a scale ranging from 'not true at all' to 'completely true'.

	Not true at all (1)	Barely true (2)	Somewhat true (3)	Completely true (4)
I can concentrate on one activity for a long time, if necessary. (1)	○	○	○	○
If I am distracted from an activity, I don't have any problem coming back to the topic quickly. (2)	○	○	○	○
If an activity arouses my feelings too much, I can calm myself down so that I can continue with the activity soon. (3)	○	○	○	○
If an activity requires a problem-oriented attitude, I can control my feelings. (4)	○	○	○	○
It is difficult for me to suppress thoughts that interfere with what I need to do. (5)	○	○	○	○
I can control my thoughts from distracting me from the task at hand. (6)	○	○	○	○

When I worry about something, I cannot concentrate on an activity. (7)	○	○	○	○
After an interruption, I don’t have any problem resuming my concentrated style of working. (8)	○	○	○	○
I have a whole bunch of thoughts and feelings that interfere with my ability to work in a focused way. (9)	○	○	○	○
I stay focused on my goal and don’t allow anything to distract me from my plan of action. (10)	○	○	○	○

Q46 Here, we would like to ask you about your resilience. For that, please indicate the extent to which you agree with each of the following statements by using a scale ranging from 'strongly disagree' to 'strongly agree':

	Strongly disagree (1)	Disagree (2)	Neutral (3)	Agree (4)	Strongly agree (5)
I tend to bounce back quickly after hard times. (1)	○	○	○	○	○
I have a hard time making it through stressful events. (2)	○	○	○	○	○
It does not take me long to recover from a stressful event. (3)	○	○	○	○	○
It is hard for me to snap back when something bad happens. (4)	○	○	○	○	○
I usually come through difficult times with little trouble. (5)	○	○	○	○	○
I tend to take a long time to get over set-backs in my life. (6)	○	○	○	○	○

Q47 For practically everyone, the corona crisis has also been a personal crisis. The next questions regard how your personal resources grew not despite, but because you experienced this crisis. Therefore, please indicate for each of the statements below the degree to which this change occurred in your life as a result of the corona crisis, using a scale ranging from 'As a result of the corona crisis, I did not experience this change' to 'As a result of the corona crisis, I experienced this change to a very great degree'.

As a result of the corona crisis,...

	I did not experience this change (1)	I experienced this change to a very small degree (2)	I experienced this change to a small degree (3)	I experienced this change to a moderate degree (4)	I experienced this change to a great degree (5)	I experienced this change to a very great degree (6)
I changed my priorities about what is important in life. (1)	○	○	○	○	○	○
I have a greater appreciation for the value of my own life. (2)	○	○	○	○	○	○
I am able to do better things with my life. (3)	○	○	○	○	○	○
I have a better understanding of spiritual matters. (4)	○	○	○	○	○	○
I have a greater sense of closeness with others. (5)	○	○	○	○	○	○
I established a new path for my life. (6)	○	○	○	○	○	○

I know better that I can handle difficulties. (7)	○	○	○	○	○	○
I have a stronger religious faith. (8)	○	○	○	○	○	○
I discovered that I'm stronger than I thought I was. (9)	○	○	○	○	○	○
I learned a great deal about how wonderful people are. (10)	○	○	○	○	○	○

Q48 In the following, we would like to ask you how you react to unforeseen events in general. Please indicate on a scale ranging from 'not at all characteristic for me' to 'entirely characteristic for me', how the following statements apply to you.

	Not at all characteristic for me (1)	Slightly characteristic for me (2)	Moderately characteristic for me (3)	Mostly characteristic for me (4)	Entirely characteristic for me (5)
Unforeseen events upset me greatly. (1)	○	○	○	○	○
It frustrates me not having all the information I need. (2)	○	○	○	○	○
One should always look ahead so as to avoid surprises. (3)	○	○	○	○	○
A small unforeseen event can spoil everything, even with the best planning. (4)	○	○	○	○	○
I always want to know what the future has in store for me. (5)	○	○	○	○	○
I can’t stand being taken by surprise. (6)	○	○	○	○	○

I should be able to organize everything in advance. (7)	o	o	o	o	o
Uncertainty keeps me from living a full life. (8)	o	o	o	o	o
When it’s time to act, uncertainty paralyses me. (9)	o	o	o	o	o
When I am uncertain, I can’t function very well. (10)	o	o	o	o	o
The smallest doubt can stop me from acting. (11)	o	o	o	o	o
I must get away from all uncertain situations. (12)	o	o	o	o	o

End of Block: Individual Predictors II

Start of Block: Learning environment-related factors

Q49 The next statements tap into your experiences during the last two weeks of studying within your learning environment. By ***learning environment***, we mean your academic

surroundings during studying. This could include interactions with your fellow students, your teachers, your mentor/coach group or people in the physical places you go to when studying. However, it also includes structural factors, such as the structure your learning environment is giving you, or specific characteristics associated with online or offline education. Please indicate for each of the statements to what extent they are true for you, using a scale ranging from 'not at all true' to 'totally true'.

During the past two weeks of studying in my learning environment...

	Not at all true (1)	Rather not true (2)	Sometimes true/sometimes not true (3)	Rather true (4)	Totally true (5)
I feel a sense of choice and freedom in the things I undertake. (1)	○	○	○	○	○
Most of the things I do feel like “I have to”. (2)	○	○	○	○	○
I feel that the people care about me. (3)	○	○	○	○	○
I feel excluded from the group of people. (4)	○	○	○	○	○
I feel confident that I can do things well. (5)	○	○	○	○	○
I have serious doubts about whether I can do things well. (6)	○	○	○	○	○
I feel that my decisions reflect what I really want. (7)	○	○	○	○	○
I feel forced to do many things I wouldn’t choose to do. (8)	○	○	○	○	○

I feel connected with people who care for me, and for whom I care. (9)	○	○	○	○	○
I feel that people who are important to me are cold and distant towards me. (10)	○	○	○	○	○
I feel capable at what I do. (11)	○	○	○	○	○
I feel disappointed with many of my performances. (12)	○	○	○	○	○
I feel my choices express who I really am. (13)	○	○	○	○	○
I feel pressured to do too many things. (14)	○	○	○	○	○
I feel close and connected with other people who are important to me. (15)	○	○	○	○	○

I have the impression that people I spend time with dislike me. (16)	○	○	○	○	○
I feel competent to achieve my goals. (17)	○	○	○	○	○
I feel insecure about my abilities. (18)	○	○	○	○	○
I feel I have been doing what really interests me. (19)	○	○	○	○	○
My daily activities feel like a chain of obligations. (20)	○	○	○	○	○
I experience a warm feeling with the people I spend time with. (21)	○	○	○	○	○
I feel the relationships I have are just superficial. (22)	○	○	○	○	○
I feel I can successfully complete difficult tasks. (23)	○	○	○	○	○

I feel like a failure because of the mistakes I make. (24)	○	○	○	○	○

Q50 Lastly, we would like to ask you some final questions about how you experienced studying in the last two weeks of studying. Here, you can answer as openly as you want to give us a short glimpse in your experiences of studying in times of a pandemic.
How would you describe the interaction between you and your teachers?

____

____

____

____

____

Q51 How would you describe the interaction between you and your fellow students?

____

____

____

____

____

Q52 Which positive and negative insights do you take with you from this period?
Positive:

____

____

____

____

____

Q54 Negative:

____

____

____

____

____

Q53 If you could help us shape the “new normal” in the educational sector, what would you want it to look like?

____

____

____

____

____

End of Block: Learning environment-related factors

Start of Block: End of Questionnaire

Q55 You have now reached the end of the questionnaire!

Q54 Do you have any questions or remarks on this questionnaire?

____

Q56 Thank you so much for your time and input, we appreciate that very, very much. As promised, we want to provide you with some additional information on how to maintain your psychological well-being in times of crisis. You can download a PDF with additional resources here ! If you have any questions regarding the questionnaire or are interested in receiving insights into the results, you are free to contact the research team [...] On the next page, you can close the questionnaire.

End of Block: End of Questionnaire
