# Supplementary material for: Student well-being in times of COVID-19 in the Netherlands: basic psychological need satisfaction and frustration within the academic learning environment
Source: Eur J Psychol Educ. 2023 Mar 2:1–21. Online ahead of print. doi: 10.1007/s10212-023-00680-x (PMC9977641; doi:10.1007/s10212-023-00680-x)
Supplement: Supplementary file 4 — Supplementary file4 (PDF 448 KB) [file 10212_2023_680_MOESM4_ESM.pdf]

Supplementary Material IV

Syntax

1. DATA PREPARATION

Explanation:

WHO5 = WHO-5 Well-Being Index (World Health Organization, 1998)

PANAS = Positive And Negative Schedule (PANAS; Watson et al., 1988)

BPNSNF = Basic Psychological Need Satisfaction and Frustration Scale (Chen et al., 2015)

1.1 Prepare data set

**Dropouts -> who filled in less than half will be excluded

-> has to be executed every time when opening the file

variable is copied in new variable 'Dropout' to calculate frequencies

```
COMPUTE
filter_$=(NMISS(WHO5_1,WHO5_2,WHO5_3,WHO5_4,WHO5_5,PANAS_1,PANAS_2,PANAS_3,PANAS_4,PANAS_5,PANAS_6,PANAS_7,

PANAS_8,PANAS_9,PANAS_10,PANAS_11,PANAS_12,PANAS_13,PANAS_14,PANAS_15,PANAS_16,PANAS_17,PANAS_18,PANAS_19,PANAS_20,

BPNSNF_1,BPNSNF_2,BPNSNF_3,BPNSNF_4,BPNSNF_5,BPNSNF_6,BPNSNF_7,BPNSNF_8,BPNSNF_9,BPNSNF_10,BPNSNF_11,BPNSNF_12,BPNSNF_13,BPNSNF_14,BPNSNF_15,

BPNSNF_16,BPNSNF_17,BPNSNF_18,BPNSNF_19,BPNSNF_20,BPNSNF_21,BPNSNF_22,BPNSNF_23,BPNSNF_24) < 49).
VARIABLE LABELS filter_$
'NMISS(WHO5_1,WHO5_2,WHO5_3,WHO5_4,WHO5_5,PANAS_1,PANAS_2,PANAS_3,PANAS_4,PANAS_5,'+

'PANAS_6,PANAS_7,PANAS_8,PANAS_9,PANAS_10,PANAS_11,PANAS_12,PANAS_13,PANAS_14,PANAS_15,PANAS_16,PANAS_17,PANAS_18,PANAS_19,'+
 'PANAS_20,BPNSNF_1, BPNSNF _2, BPNSNF _3, BPNSNF _4, BPNSNF _5, BPNSNF _6, BPNSNF _7, BPNSNF _8, BPNSNF _9, BPNSNF _10, BPNSNF _11, BPNSNF _12... (FILTER)'.
```

```
VALUE LABELS filter_$ 0 'Not Selected' 1 'Selected'.

FORMATS filter_$ (f1.0).

FILTER BY filter_$.

EXECUTE.


FREQUENCIES VARIABLES=Dropout

  /ORDER=ANALYSIS.


*if analysis should be run including dropouts:


FILTER OFF.

USE ALL.

EXECUTE.


**Flatlining for every variable

only participants flatlining at scales with more than 10 items will be excluded

SDs are calculated and recoded (SD = 0 -> 0, SD > 0 -> 1)

everyone with a 0 will be excluded

-> n(Flatliners) = 10; are excluded by hand


COMPUTE SD_WHO=SD(WHO5_1,WHO5_2,WHO5_3,WHO5_4,WHO5_5).

COMPUTE
SD_PANAS=SD(PANAS_1,PANAS_2,PANAS_3,PANAS_4,PANAS_5,PANAS_6,PANAS_7,PANAS_8,PANAS_9,PANAS_10,PANAS_11,PANAS_12,

PANAS_13,PANAS_14,PANAS_15,PANAS_16,PANAS_17,PANAS_18,PANAS_19,PANAS_20).

COMPUTE

SD_BPN=SD(BPNSNF_1,BPNSNF_2,BPNSNF_3,BPNSNF_4,BPNSNF_5,BPNSNF_6,BPNSNF_7,BPNSNF_8,BPNSNF_9,BPNSNF_10,BPNSNF_11,BPNSNF_12,

BPNSNF_13,BPNSNF_14,BPNSNF_15,BPNSNF_16,BPNSNF_17,BPNSNF_18,BPNSNF_19,BPNSNF_20,BPNSNF_21,BPNSNF_22,BPNSNF_23,BPNSNF_24).

EXECUTE.
```

```
RECODE SD_WHO (MISSING=SYSMIS) (0=0) (ELSE=1)..
RECODE SD_PANAS (MISSING=SYSMIS) (0=0) (ELSE=1).
RECODE SD_BPN (MISSING=SYSMIS) (0=0) (ELSE=1).
EXECUTE.

FREQUENCIES VARIABLES=SD_WHO SD_PANAS SD_BPN
  /ORDER=ANALYSIS.
```

1.2. Calculating means

```
**WHO-5 calculating, descriptives, & resiliability

COMPUTE WHO5=SUM(WHO5_1,WHO5_2,WHO5_3,WHO5_4,WHO5_5)-5.
EXECUTE.

DESCRIPTIVES VARIABLES=WHO5
  /STATISTICS=MEAN STDDEV MIN MAX.

RELIABILITY
  /VARIABLES=WHO5_1,WHO5_2,WHO5_3,WHO5_4,WHO5_5
  /SCALE('ALL VARIABLES') ALL
  /MODEL=ALPHA
  /STATISTICS=DESCRIPTIVE SCALE CORR
  /SUMMARY=TOTAL.

*Below 13 = poor well-being -> dichotomous WHO-5; recoding & descriptives

RECODE WHO5 (MISSING=SYSMIS) (0 thru 12=1) (13 thru 25=2) INTO WHO5_dich.
VARIABLE LABELS  WHO5_dich 'WHO5 dichotomous'.
```

```
EXECUTE.

FREQUENCIES VARIABLES=WHO5_dich
  /ORDER=ANALYSIS.

**PANAS
PA = positive affect; NA = negative affect
calculating, descriptives, & reliabilities

COMPUTE
PA=SUM(PANAS_1,PANAS_2,PANAS_3,PANAS_4,PANAS_5,PANAS_6,PANAS_7,PANAS_8,PANAS_9,PANAS_10).
EXECUTE.

COMPUTE
NA=SUM(PANAS_11,PANAS_12,PANAS_13,PANAS_14,PANAS_15,PANAS_16,PANAS_17,PANAS_18,PANAS_19,PANAS_20).
EXECUTE.

DESCRIPTIVES VARIABLES=PA NA
  /STATISTICS=MEAN STDDEV MIN MAX.

RELIABILITY

/VARIABLES=PANAS_1,PANAS_2,PANAS_3,PANAS_4,PANAS_5,PANAS_6,PANAS_7,PANAS_8,PANAS_9,PANAS_10
  /SCALE('ALL VARIABLES') ALL
  /MODEL=ALPHA
  /STATISTICS=DESCRIPTIVE SCALE CORR
  /SUMMARY=TOTAL.

RELIABILITY
```

```
/VARIABLES=PANAS_11,PANAS_12,PANAS_13,PANAS_14,PANAS_15,PANAS_16,PANAS_17,PANAS_18,PANAS_19,PANAS_20
  /SCALE('ALL VARIABLES') ALL
  /MODEL=ALPHA
  /STATISTICS=DESCRIPTIVE SCALE CORR
  /SUMMARY=TOTAL.
```

**BPN

Aut = Autonomy, Com = Competence, Rel = Relatedness; S = Satisfaction, F = Frustration

Calculating facets, descriptives, & reliabilities (for subscales)

```
COMPUTE AutS=MEAN(BPNSNF_1,BPNSNF_7,BPNSNF_13,BPNSNF_19).
COMPUTE AutF=MEAN(BPNSNF_2,BPNSNF_8,BPNSNF_14,BPNSNF_20).
COMPUTE ComS=MEAN(BPNSNF_5,BPNSNF_11,BPNSNF_17,BPNSNF_23).
COMPUTE ComF=MEAN(BPNSNF_6,BPNSNF_12,BPNSNF_18,BPNSNF_24).
COMPUTE RelS=MEAN(BPNSNF_3,BPNSNF_9,BPNSNF_15,BPNSNF_21).
COMPUTE RelF=MEAN(BPNSNF_4,BPNSNF_10,BPNSNF_16,BPNSNF_22).
EXECUTE.

DESCRIPTIVES VARIABLES=AutS AutF ComS ComF RelS RelF
  /STATISTICS=MEAN STDDEV MIN MAX.

RELIABILITY
  /VARIABLES=BPNSNF_1,BPNSNF_7,BPNSNF_13,BPNSNF_19
  /SCALE('ALL VARIABLES') ALL
  /MODEL=ALPHA
  /STATISTICS=DESCRIPTIVE SCALE CORR
  /SUMMARY=TOTAL.

RELIABILITY
  /VARIABLES=BPNSNF_2,BPNSNF_8,BPNSNF_14,BPNSNF_20
```

```
 /SCALE('ALL VARIABLES') ALL
 /MODEL=ALPHA
 /STATISTICS=DESCRIPTIVE SCALE CORR
 /SUMMARY=TOTAL.

RELIABILITY
 /VARIABLES=BPNSNF_5,BPNSNF_11,BPNSNF_17,BPNSNF_23
 /SCALE('ALL VARIABLES') ALL
 /MODEL=ALPHA
 /STATISTICS=DESCRIPTIVE SCALE CORR
 /SUMMARY=TOTAL.

RELIABILITY
 /VARIABLES=BPNSNF_6,BPNSNF_12,BPNSNF_18,BPNSNF_24
 /SCALE('ALL VARIABLES') ALL
 /MODEL=ALPHA
 /STATISTICS=DESCRIPTIVE SCALE CORR
 /SUMMARY=TOTAL.

RELIABILITY
 /VARIABLES=BPNSNF_3,BPNSNF_9,BPNSNF_15,BPNSNF_21
 /SCALE('ALL VARIABLES') ALL
 /MODEL=ALPHA
 /STATISTICS=DESCRIPTIVE SCALE CORR
 /SUMMARY=TOTAL.

RELIABILITY
 /VARIABLES=BPNSNF_4,BPNSNF_10,BPNSNF_16,BPNSNF_22
 /SCALE('ALL VARIABLES') ALL
 /MODEL=ALPHA
 /STATISTICS=DESCRIPTIVE SCALE CORR
```

```
  /SUMMARY=TOTAL.


*factor analysis

first according to eigenvalue = 1; second fixed factors =3


FACTOR

  /VARIABLES BPNSNF_1 BPNSNF_2 BPNSNF_3 BPNSNF_4 BPNSNF_5 BPNSNF_6 BPNSNF_7 BPNSNF_8 BPNSNF_9 BPNSNF_10 BPNSNF_11 BPNSNF_12 BPNSNF_13

    BPNSNF_14 BPNSNF_15 BPNSNF_16 BPNSNF_17 BPNSNF_18 BPNSNF_19 BPNSNF_20 BPNSNF_21 BPNSNF_22 BPNSNF_23 BPNSNF_24

  /MISSING LISTWISE

  /ANALYSIS BPNSNF_1 BPNSNF_2 BPNSNF_3 BPNSNF_4 BPNSNF_5 BPNSNF_6 BPNSNF_7 BPNSNF_8 BPNSNF_9 BPNSNF_10 BPNSNF_11 BPNSNF_12 BPNSNF_13

    BPNSNF_14 BPNSNF_15 BPNSNF_16 BPNSNF_17 BPNSNF_18 BPNSNF_19 BPNSNF_20 BPNSNF_21 BPNSNF_22 BPNSNF_23 BPNSNF_24

  /PRINT UNIVARIATE INITIAL CORRELATION KMO REPR AIC EXTRACTION ROTATION

  /FORMAT SORT

  /PLOT EIGEN

  /CRITERIA MINEIGEN(1) ITERATE(25)

  /EXTRACTION ML

  /CRITERIA ITERATE(25) DELTA(0)

  /ROTATION OBLIMIN.


FACTOR

  /VARIABLES BPNSNF_1 BPNSNF_2 BPNSNF_3 BPNSNF_4 BPNSNF_5 BPNSNF_6 BPNSNF_7 BPNSNF_8 BPNSNF_9 BPNSNF_10 BPNSNF_11 BPNSNF_12 BPNSNF_13

    BPNSNF_14 BPNSNF_15 BPNSNF_16 BPNSNF_17 BPNSNF_18 BPNSNF_19 BPNSNF_20 BPNSNF_21 BPNSNF_22 BPNSNF_23 BPNSNF_24

  /MISSING LISTWISE

  /ANALYSIS BPNSNF_1 BPNSNF_2 BPNSNF_3 BPNSNF_4 BPNSNF_5 BPNSNF_6 BPNSNF_7 BPNSNF_8 BPNSNF_9 BPNSNF_10 BPNSNF_11 BPNSNF_12 BPNSNF_13

    BPNSNF_14 BPNSNF_15 BPNSNF_16 BPNSNF_17 BPNSNF_18 BPNSNF_19 BPNSNF_20 BPNSNF_21 BPNSNF_22 BPNSNF_23 BPNSNF_24

  /PRINT UNIVARIATE INITIAL CORRELATION KMO REPR AIC EXTRACTION ROTATION
```

```
  /FORMAT SORT
  /PLOT EIGEN
  /CRITERIA FACTORS(3) ITERATE(25)
  /EXTRACTION ML
  /CRITERIA ITERATE(25) DELTA(0)
  /ROTATION OBLIMIN.
```

3. ANALYSIS

3.1 Correlations

*preparation: Bachelor/Master students recode to dichotomous, excluding pre-Masters, as they make up only 4% and are not internationally comparable

```
RECODE studies (1=1) (3=3) (2.4=SYSMIS) (MISSING=SYSMIS) INTO BA_MA.
VARIABLE LABELS  BA_MA 'Bachelor vs Master students'.
EXECUTE.
```

*Pearson correlations with interval-scaled variables: PA & NA, WHO, need satisfaction & frustration, age

with first checking for normal distribution of the

```
EXAMINE VARIABLES=PA NA WHO5 AutS AutF ComS ComF RelS RelF age
  /PLOT BOXPLOT HISTOGRAM NPPLOT
  /COMPARE GROUPS
  /STATISTICS DESCRIPTIVES
  /CINTERVAL 95
  /MISSING LISTWISE
  /NOTOTAL.
```

*Conclusion: based on the histogram, NA, RelS, RelF & age will not be considered as normally distributed and, hence, included in Spearmans r

Additonally, the outliers displayed in the output were deleted from the data set

*additional to Pearson also Point-biserial correlation with one dichotomous and one interval-scaled variable: PA, WHO, need satisfaction & frustration (except Rel),

gender, bachelor/master, international, firstgen, and living alone; Because Point-biserial is a special case of Pearson

```
CORRELATIONS
  /VARIABLES=WHO5 PA AutS AutF ComS ComF Gender BA_MA International firstgeneration_2 livingsituation_1
  /PRINT=TWOTAIL NOSIG
  /STATISTICS DESCRIPTIVES
  /MISSING=PAIRWISE.
```

*Spearman correlations with ordinal-scaled variables: additional to the above: NA, Rel, age

```
NONPAR CORR
/VARIABLES=WHO5 PA AutS AutF ComS ComF Gender BA_MA International Firstgeneration_2 LIVINGSITUATION_1 NA RelS RelF age
/PRINT=SPEARMAN TWOTAIL NOSIG
```

3.3. ANOVAs

**MANOVA to detect potentiall differences for well-being and need variables based on faculty affiliation:

```
GLM WHO5 PA NA AutS AutF ComS ComF RelS RelF BY Faculty
  /METHOD=SSTYPE(3)
  /INTERCEPT=INCLUDE
  /POSTHOC=Faculty(LSD)
  /PRINT=DESCRIPTIVE ETASQ OPOWER HOMOGENEITY
  /CRITERIA=ALPHA(.05)
  /DESIGN= Faculty.
```

3.4 Multiple Regression

*multiple regression with BPN as predictors, gender, international, living alone, first gen age, faculty as control, & WHO outcome variable

```
REGRESSION
  /DESCRIPTIVES MEAN STDDEV CORR SIG N
  /MISSING LISTWISE
  /STATISTICS COEFF OUTS CI(95) R ANOVA COLLIN TOL CHANGE ZPP
  /CRITERIA=PIN(.05) POUT(.10)
  /NOORIGIN
  /DEPENDENT WHO5
  /METHOD=ENTER Gender International Livingsituation_1 Firstgeneration_2 age dum_BSS dum_UMCG dum_FSS dum_other dum_2faculties
  /METHOD=ENTER Gender International livingsituation_1 Firstgeneration_2 age dum_BSS dum_UMCG dum_FSS dum_other dum_2faculties AutS AutF ComS ComF RelS RelF
  /SCATTERPLOT=(*ZRESID ,*ZPRED)
  /RESIDUALS DURBIN HISTOGRAM(ZRESID) NORMPROB(ZRESID)
  /CASEWISE PLOT(ZRESID) OUTLIERS(2)
  /SAVE PRED ZPRED ADJPRED MAHAL COOK LEVER ZRESID DRESID SDRESID SDBETA SDFIT COVRATIO.
```

*multiple regression with BPN as predictors, gender, international, times on campus, academic delay & workload as control, & PA outcome variable

```
REGRESSION
  /DESCRIPTIVES MEAN STDDEV CORR SIG N
  /MISSING LISTWISE
  /STATISTICS COEFF OUTS CI(95) R ANOVA COLLIN TOL CHANGE ZPP
  /CRITERIA=PIN(.05) POUT(.10)
  /NOORIGIN
  /DEPENDENT PA
```

/METHOD=ENTER Gender International livingsituation_1 Firstgeneration_2 age dum_BSS dum_UMCG dum_FSS dum_other dum_2faculties

/METHOD=ENTER Gender International livingsituation_1 Firstgeneration_2 age dum_BSS dum_UMCG dum_FSS dum_other dum_2faculties AutS AutF ComS ComF RelS RelF

/SCATTERPLOT=(*ZRESID ,*ZPRED)

/RESIDUALS DURBIN HISTOGRAM(ZRESID) NORMPROB(ZRESID)

/CASEWISE PLOT(ZRESID) OUTLIERS(2)

/SAVE PRED ZPRED ADJPRED MAHAL COOK LEVER ZRESID DRESID SDRESID SDBETA SDFIT COVRATIO.

*multiple regression with BPN as predictors, gender, international, times on campus, academic delay & workload as control, & NA outcome variable

REGRESSION

/DESCRIPTIVES MEAN STDDEV CORR SIG N

/MISSING LISTWISE

/STATISTICS COEFF OUTS CI(95) R ANOVA COLLIN TOL CHANGE ZPP

/CRITERIA=PIN(.05) POUT(.10)

/NOORIGIN

/DEPENDENT NA

/METHOD=ENTER Gender International livingsituation_1 Firstgeneration_2 age dum_BSS dum_UMCG dum_FSS dum_other dum_2faculties

/METHOD=ENTER Gender International livingsituation_1 Firstgeneration_2 age dum_BSS dum_UMCG dum_FSS dum_other dum_2faculties AutS AutF ComS ComF RelS RelF

/SCATTERPLOT=(*ZRESID ,*ZPRED)

/RESIDUALS DURBIN HISTOGRAM(ZRESID) NORMPROB(ZRESID)

/CASEWISE PLOT(ZRESID) OUTLIERS(2)

/SAVE PRED ZPRED ADJPRED MAHAL COOK LEVER ZRESID DRESID SDRESID SDBETA SDFIT COVRATIO.
